# Multipronged Therapeutic Effects of Chinese Herbal Medicine Qishenyiqi in the Treatment of Acute Myocardial Infarction

**DOI:** 10.3389/fphar.2017.00098

**Published:** 2017-03-02

**Authors:** Yong Wang, Weili Lin, Chun Li, Sarita Singhal, Gaurav Jain, Lixin Zhu, Linghui Lu, Ruixin Zhu, Wei Wang

**Affiliations:** ^1^Department of Biochemistry and Molecular Biology, Basic Medical College, Beijing University of Chinese MedicineBeijing, China; ^2^Key Lab of Computational Biology, CAS-MPG Partner Institute for Computational Biology, Shanghai Institutes for Biological Sciences, Chinese Academy of SciencesShanghai, China; ^3^Modern Research Center for Traditional Chinese Medicine, Beijing University of Chinese MedicineBeijing, China; ^4^Department of Pediatrics, Digestive Diseases and Nutrition Center, State University of New York at BuffaloBuffalo, NY, USA; ^5^Niagara Falls Memorial Medical Center and Invision HealthBuffalo, NY, USA; ^6^Genome, Environment and Microbiome Community of Excellence, State University of New York at BuffaloBuffalo, NY, USA; ^7^Institute of Digestive Diseases, Longhua Hospital, Shanghai University of Traditional Chinese MedicineShanghai, China; ^8^Department of Bioinformatics, School of Life Sciences and Technology, Tongji UniversityShanghai, China

**Keywords:** Acute myocardial infarction, Qishenyiqi, anti-inflammation, dyslipidaemia, group significance analysis

## Abstract

**Background:** Based on global gene expression profile, therapeutic effects of Qishenyiqi (QSYQ) on acute myocardial infarction (AMI) were investigated by integrated analysis at multiple levels including gene expression, pathways involved and functional group.

**Methods:** Sprague-Dawley (SD) rats were randomly divided into 3 groups: Sham-operated, AMI model (left anterior descending coronary artery ligation) and QSYQ-treated group. Cardiac tissues were obtained for analysing digital gene expression. Sequencing and transcriptome analyses were performed collaboratively, including analyses of differential gene expression, gene co-expression network, targeted attack on network and functional grouping. In this study, a new strategy known as keystone gene-based group significance analysis was also developed.

**Results:** Analysis of top keystone QSYQ-regulated genes indicated that QSYQ ameliorated ventricular remodeling (VR), which is an irreversible process in the pathophysiology of AMI. At pathway level, both well-known cardiovascular diseases and cardiac signaling pathways were enriched. The most remarkable finding was the novel therapeutic effects identified from functional group analysis. This included anti-inflammatory effects mediated via suppression of arachidonic acid lipoxygenase (LOX) pathway and elevation of nitric oxide (NO); and amelioration of dyslipidaemia mediated via fatty acid oxidation. The regulatory patterns of QSYQ on key genes were confirmed by western blot, immunohistochemistry analysis and measurement of plasma lipids, which further validated the therapeutic effects of QSYQ proposed in this study.

**Conclusions:** QSYQ exerts multipronged therapeutic effects on AMI, by concurrently alleviating VR progression, attenuating inflammation induced by arachidonic acid LOX pathway and NO production; and ameliorating dyslipidaemia.

## Introduction

Acute myocardial infarction (AMI) is the most frequent cause of heart failure (HF) (Mahmood et al., 2014; Maranhão and Tavares, 2015). Although evidence-based therapies have been established, AMI-induced HF remains a leading cause of global mortality (Gaziano et al., 2006). Herbal formulas of traditional Chinese medicine (TCM) have been effectively used in treatment of AMI with few side-effects (Ferreira and Lopes, 2011). One of the commonly used TCM for AMI is Qishenyiqi (QSYQ). QSYQ originates from the recordings of classic medical textbook, “Treatise on Exogenous Febrile Disease.” It has been effectively used in treatment of several diseases for thousands of years. It is prepared from 6 herbs, including 2 star herbs: *Astragalus membranaceus* (Fisch.) Bunge (“huang-qi” in Chinese) and *Salvia Miltiorrhiza* Bunge (“dan-shen” in Chinese), and 4 adjunctive herbs: *Lonicera japonica* Thunb., *Scrophularia aestivalis* Griseb., *Aconitum fischeri* Rchb., and *Glycyrrhiza uralensis* Fisch.

Clinical studies and preclinical researches in human and animal models have shown the positive effects of QSYQ on the ejection fraction as well as hemodynamics in AMI (Wang et al., 2012a,b, 2013; Li et al., 2014; Luo et al., 2014). Recently, extensive efforts have been made to investigate the mechanisms of action of QSYQ in the treatment of AMI. Studies on animal models have shown that QSYQ improves ligation-induced left ventricular remodeling (VR), by attenuating inflammation and fibrosis. This is mediated via interleukin 6 (IL-6)—signal transducer and activator of transcription 3 (STAT3) and tumor necrosis factor α (TNFα)—nuclear factor kappa-light-chain-enhancer of activated B cells (NFκB) signaling pathway, and this leads to decreased concentrations of transforming growth factor β (TGF-β) and matrix metallopeptidase 9 (MMP-9) (Li et al., 2014). Wang et al. (2012b) predicted potential drug targets of QSYQ using bioinformatics. They also performed experiments to validate the effects of QSYQ on these predicted targets on an animal model. One of the possible target is the inhibition of Renin-angiotensin-aldosterone system (RAAS) pathway, including renin, angiotensin II type 1 receptor (AT1), angiotensin II type 2 receptor (AT2), and angiotensin converting enzyme 2 (ACE2) (Wang et al., 2012a). Furthermore, as downstream molecules of RAAS pathway, different subtypes of nicotinamide adenine dinucleotide phosphate (NADPH) oxidase (NOX) in oxidative stress are also regulated by QSYQ in different patterns. Specific pathways including angiotensin II (AngII)—NADPH oxidase 2 (NOX2)—reactive oxygen species (ROS)—matrix metallopeptidases (MMPs) and angiotensin II (AngII)—NADPH oxidase 4 (NOX4)—reactive oxygen species (ROS)—matrix metallopeptidases (MMPs) are also regulated by QSYQ and contribute to the reduction of apoptosis in p53 and caspase-3 pathways (Wang et al., 2013). In addition, components of QSYQ also exert effects on lipid metabolism, including down-regulating plasma ox-low density lipoprotein (ox-LDL) and low density lipoprotein (LDL) levels (Yong et al., 2012). Collectively, these studies demonstrate diverse therapeutic effects of QSYQ on AMI-induced HF, including anti-inflammation, anti-fibrosis, anti-apoptosis, inhibition of RAAS activation and lipid metabolism disorder, which eventually lead to restoration of normal hemodynamic parameters and cardiac function.

Previous studies indicated that QSYQ exerts its therapeutic effects on AMI through different target pathways in a synergistic manner (Wang et al., 2012a,b, 2013; Qiu et al., 2014). However, the major limitation of these studies is that the effects of QSYQ on different pathways were not examined simultaneously. TCM is an ancient medical practice system which emphasizes the integrity of entire human body and it usually exerts therapeutic effects via multiple targets or pathways (Qiu et al., 2014). However, determination of multiple complex pathways and their interactions remains a challenge for TCM research. In recent years, multiple high-throughput omics technologies, including transcriptomics, proteomics, metabolomics and metagenomics, have been increasingly applied in TCM research (Gu and Chen, 2014). To meet the development and application of omics technologies in TCM research, routine bioinformatics strategies, which focus on differentially expressed genes (DEGs) and enriched pathways, have been evolved into advanced stages, which need to integrate multiple omics data, and more advanced network-based methods to identify hub genes and functional groups regulated by drug intervention (Berg, 2014). In our study, we obtained a well characterized high-throughput RNA sequencing expression profile (Zhu et al., 2011), complemented by Western blot, immunohistochemistry (IHC) and measurement of plasma lipids, to identify global gene expression changes induced by QSYQ treatment on AMI. We also developed a new strategy to analyse the significance of functional group based on the priorities of keystone genes, to better understand the molecular mechanisms of these gene expression changes.

## Materials and methods

### Experimental animals

Studies were performed according to the “Guide for the Care and Use of Laboratory Animals” published by National Institutes of Health (NIH Publications No. 85-23, revised 1996) and with approval of the Animal Care Committee of Beijing University of Chinese Medicine. A total of 45 male Sprague-Dawley (SD) rats with weights of 240 ± 10 g in Specific Pathogen Free (SPF) grade were selected (purchased from Beijing Vital River Laboratory Animal Technology Co. Ltd.).

### Model preparation of AMI and grouping

Total 45 rats were randomly divided into 3 groups (15 rats per group): Sham-operated, AMI model and QSYQ-treated group. Rats in AMI model and QSYQ-treated groups received ligation surgery of left anterior descending (LAD) coronary artery (Wang et al., 2012b). Left thoracotomy between third and fourth intercostal space was performed on rats. After exposing the cardiac tissues, LAD was ligated with a sterile suture (Shuangjian, Shanghai, P.R. China) 1 mm below the left atrium. The thorax was then closed layer by layer. After thoracotomy, rats were warmed on a heated blanket. One day after the surgery, rats in QSYQ-treated group received a dosage of 2.33 g/kg concentrated QSYQ via oral gavage. QSYQ is a combination of six Chinese herbs that includes *A. membranaceus* (Fisch.) Bunge, *S. Miltiorrhiza* Bunge, *L. japonica* Thunb., *S. aestivalis* Griseb., *A. fischeri* Rchb. and *G. uralensis* Fisch. in the ratios of 10:5:3:3:3:2 (Wang et al., 2012b). It was derived by mixing the residue of *A. membranaceus* (Fisch.) Bunge with all *S. Miltiorrhiza* Bunge, *L. japonica* Thunb., *S. aestivalis* Griseb., *A. fischeri* Rchb. and *G. uralensis* Fisch., followed by extraction with hot water (twice, 2 h each). The water extract was then concentrated to form a paste, and the ethanol is added. After 24 h, the filtration is collected to form the final product (Wang et al., 2012b). Quality of QSYQ was evaluated and established by its fingerprints with HPLC-IT-TOF method (shown in Figure S1). The dosage is ideal to exert definite effect for QSYQ on AMI rat model according to our previous study (Li et al., 2014). Rats in AMI model group were only treated with the same volume of water as QSYQ-treated group (Wang et al., 2012a). Rats in Sham-operated group underwent the same procedure like AMI model rats but had no actual ligation of LAD. The mortality rate of surgery was 26.7%. After surgery, there were 2, 6, and 4 rats which died inadvertently in Sham-operated, AMI model and QSYQ-treated groups respectively. Most of these rats died on the day of surgery or the following day, likely due to acute pump failure or lethal arrhythmias. After 28 days, eight rats from each group were assessed by echocardiography following overnight fasting. The cardiac tissues of all rats were harvested for subsequent molecular biology experiment (13, 9, and 11 rats in Sham-operated, AMI model, and QSYQ-treated groups respectively). Among them, 9 rats were selected randomly for digital gene expression sequencing (3 rats per group). Total RNA of cardiac tissues was extracted with TRIzol Reagent (Gibco-BRL, Paisley, UK). The concentration of RNA was measured with Nano Drop 2000 (Thermo Scientific, USA).

### Echocardiographic assessment of left ventricular (LV) function

The high-frequency, high-resolution digital imaging platform from the Vevo 2100 system (VisualSonics Inc., Canada) was applied to generate 2D M-mode and B-mode echocardiography. After 28 days of QSYQ treatment and before harvesting the cardiac tissues, 8 rats in each group were assessed by echocardiography. The independent operator was from scientific center of Beijing University of Chinese Medicine. LV internal diameter at end-diastole (LVIDd), LV internal diameter at end-systole (LVIDs), ejection fraction (EF) and fractional shortening (FS) were detected by echocardiography (Sharrett et al., 2001; Wang et al., 2015).

### Hemodynamic measurements in rats

LV performance was measured in rats after they were anesthetized with 2% isoflurane as described earlier (Li et al., 2014). A terminal hemodynamic study was performed 28 days after surgery. Nine rats were included in each group. Right carotid artery, LV systolic and end-diastolic, diastolic and mean aortic pressures, Max dP/dt, Min dP/dt, and heart rate were recorded with a system of PowerLab ML880 (AD Instrument, Australia).

### DGE-tag gene expression profiling

Cardiac tissues of ischemic marginal zone were collected after 28 days of QSYQ treatment as described above. After extracting from cardiac tissues, mRNA was purified and reverse transcribed into cDNA with Oligo (dT) magnetic beads. The cDNA was then digested with 2 endonucleases (NlaIII and MmeI) and ligated with adaptors. After linear PCR amplification of cDNA, the library of each sample was sequenced with Illumina HiSeq™ 2000. Dirty tags were filtered out. All clean tags were aligned to rat reference sequences and unambiguous tags were annotated. The number of clean tags belonging to each gene was computed as raw gene expression count, which could be normalized to the total number of transcripts in each sample per million clean tags (TPM normalization). Finally, a well-characterized transcriptome profile of total 10,515 mRNAs was established.

### Gene co-expression network (GCN) analysis

Pearson's correlation coefficient was used as co-expression measure for constructing GCN. For a given coefficient of a gene pair, Student asymptotic *p*-value was calculated and then adjusted with false discovery rate (FDR). GCN consists of gene pairs with FDR < 0.05.

PageRank centrality (Brin and Page, 2012) is an indicator to identify “hub genes” in GCN. It is the extension of degree centrality, which indicates significance of nodes in a network. PageRank, together with targeted attack on network analysis (Achard et al., 2006), was used to identify keystone QSYQ-regulated genes.

### Keystone gene-based group significance analysis

Since the enriched KEGG pathways have close linkages between each other in KEGG map, we merged them to form a background network, using KEGGgraph R package. From this network, all QSYQ-responsive genes (DEGs of comparison between QSYQ-treated and AMI model groups) were extracted to form a sub-network. Functional groups were divided from the sub-network using “leading eigenvector” method (Newman, 2006) with igraph R package. A new strategy was further developed to calculate enrichment degrees of functional groups with keystone QSYQ-regulated genes, and estimate significance levels. There are 3 key steps in this strategy.

#### Step 1: generation of priority list for keystone QSYQ-regulated genes

PageRank scores in GCN were scaled in the range of 0–1. The minimum of PageRank scores was subtracted from each PageRank score. Each PageRank score was then divided by the range of PageRank scores. A priority list L consists of scaled PageRank scores of keystone QSYQ-regulated genes in sub-network. Genes in list L were prioritized in decreasing order of scaled PageRank scores.

#### Step 2: calculation of enrichment score (ES) for each functional group

We calculated ES that reflects the degree to which a functional group C is overrepresented at the top of priority list L. Given the list L, ES was calculated by walking down list L. During the walking, when we encounter a gene in group C, we increase a running-sum statistic (hits score “*P*_*hit*_”); when we encounter a gene not in group C, we decrease it (penalty score “*P*_*miss*_”). “*P*_*hit*_” and “*P*_*miss*_” were calculated as formulas (1) and (2), respectively. In the formula (1), s_*j*_ represents scaled PageRank score of gene g_*j*._ In the formula (2), *N*_*diff*_ means the count of genes in difference set between group C and list L; *N*_*genelist*_ and *N*_*group*_ denote the count of genes in list L and group C, respectively.

(1) $$P_{hit}(C,i)=\sum_{\begin{array}{l} g_{i}\in C\\ \text{j }\leq \;i \end{array}}S_{j}$$

(2) $$P_{miss}(C,i)=\sum_{\begin{array}{l} g_{j}\notin C\\ j\;\leq \;i \end{array}}\frac{N_{diff}}{N_{genelist}\times N_{group}}$$

Finally, ES is the maximum of *P*_*hit*_-*P*_*miss*_. If genes in a group C are randomly distributed across list L, or distributed at the bottom of list L, ES(C) will be relatively low or even negative. On the contrary, if they are concentrated at the top of list L, ES(C) will be high. In our strategy, both count (*P*_*miss*_) and significance degree (*P*_*hit*_) of keystone genes were taken into considerations.

#### Step 3: estimation of significance level of ES by monte carlo simulation

For a functional group C, the statistical significance (nominal P value) of ES was estimated over a null distribution, which was generated from 10,000 times of Monte Calro simulations. The main steps are as follows:

- Randomly sample N_*C*_ genes from the background network to form a group C_random_ and re-compute ES_random_. N_*C*_ is the count of genes in group C,
- Repeat step (a) for 10,000 times, and create a null distribution of enrichment scores ES_NULL_,
- Estimate nominal P value for ES of group C from ES_NULL_ with the positive portion, that is P(C) = #(ES_NULL_ ≥ observed ES)/10,000, which #(ES_NULL ≥_ observedES) represents the count of ES_NULL_ that is larger than the ES of group C.

The estimated significance levels (*P*-values) of all the functional groups were adjusted for multiple hypothesis testing with Simes-Hochberg method (Jacquez, 1996).

### Experimental validation with western blot, IHC and measurement of plasma lipids (TC, TG, HDL, and LDL)

Cardiac tissues were lysed and homogenized using RIPA buffer (Applygen Technologies Inc., Beijing, China). Total protein was extracted from the tissue homogenate. Concentration in each sample was detected by BCA assay kit (Applygen Technologies Inc., Beijing, China). Equal amounts of protein were separated with SDS-PAGE and transferred onto NC membranes (Applygen Technologies Inc., Beijing, China). The following primary antibodies were used: MMP-2(1:500 dilution; Abcam, Cambridge, UK), NOS3(1:2,500 dilution; BD Transduction Laboratories, US), ALOX15(1:1,000 dilution; Abcam, Cambridge, UK), CPT2(1:1,000 dilution; Abcam, Cambridge, UK), CD36(1:1000 dilution; Abcam, Cambridge, UK), LPL(1:500 dilution; Abcam, Cambridge, UK), BCKDHA(1:1,000 dilution; Abcam, Cambridge, UK), DBT(1:500 dilution; Abcam, Cambridge, UK), GAPDH(1:10,000 dilution; Abcam, Cambridge, UK). Three rats in each group were detected for 3 biological replicates.

IHC was performed with cell & tissue staining kits (R&D Systems, Inc., USA) and followed by the protocol (Li et al., 2014). 6 rats in each group were incubated with primary antibody (1:200 dilution; Anti-Collagen III antibody, Abcam, Cambridge, UK; 1:200 dilution; Anti-Collagen V antibody, Abcam, Cambridge, UK) for 12 h. 5 visual fields were selected for each sample and analyzed with Image-Pro Plus 6.0 software to determine the integrated optical density (IOD) in the positive stained areas.

Twenty-eight days after surgery, animals were sacrificed after anaesthetization and blood was taken through abdominal aorta. The blood was centrifuged at 8,000 g for 10 min and supernatant was used for detection of plasma indicators. Six rats were included in each group. Plasma levels of total cholesterol (TC), triglyceride (TG), high density lipoprotein (HDL) and low density lipoprotein (LDL) were measured by automatic biochemical analyzer (HITACH17080, Japan). Plasma levels were quantified by comparing with standards of TC, TG, HDL, and LDL from Sekisui chemical Diagnostic reagents (Sekisui chemical co., LTD, Japan).

### Statistical analysis

DEGs were detected by DESeq2 R package, with FDR < 0.10 (for reference of Love et al., 2014) and absolute value of log2 fold change >0.58. KEGG enrichment analysis was performed using “clusterProfiler” R package with *P* < 0.05. In experimental validation, all data were presented as mean ± standard deviation. Statistical analysis was carried out on one-way analysis of variance (ANOVA) and Dunnetts' test. *P* < 0.05 were considered statistically significant. Statistical analysis was performed in R 3.1.3 software.

## Results

### Model evaluation by echocardiography and hemodynamic measurements

**Figure 1:**
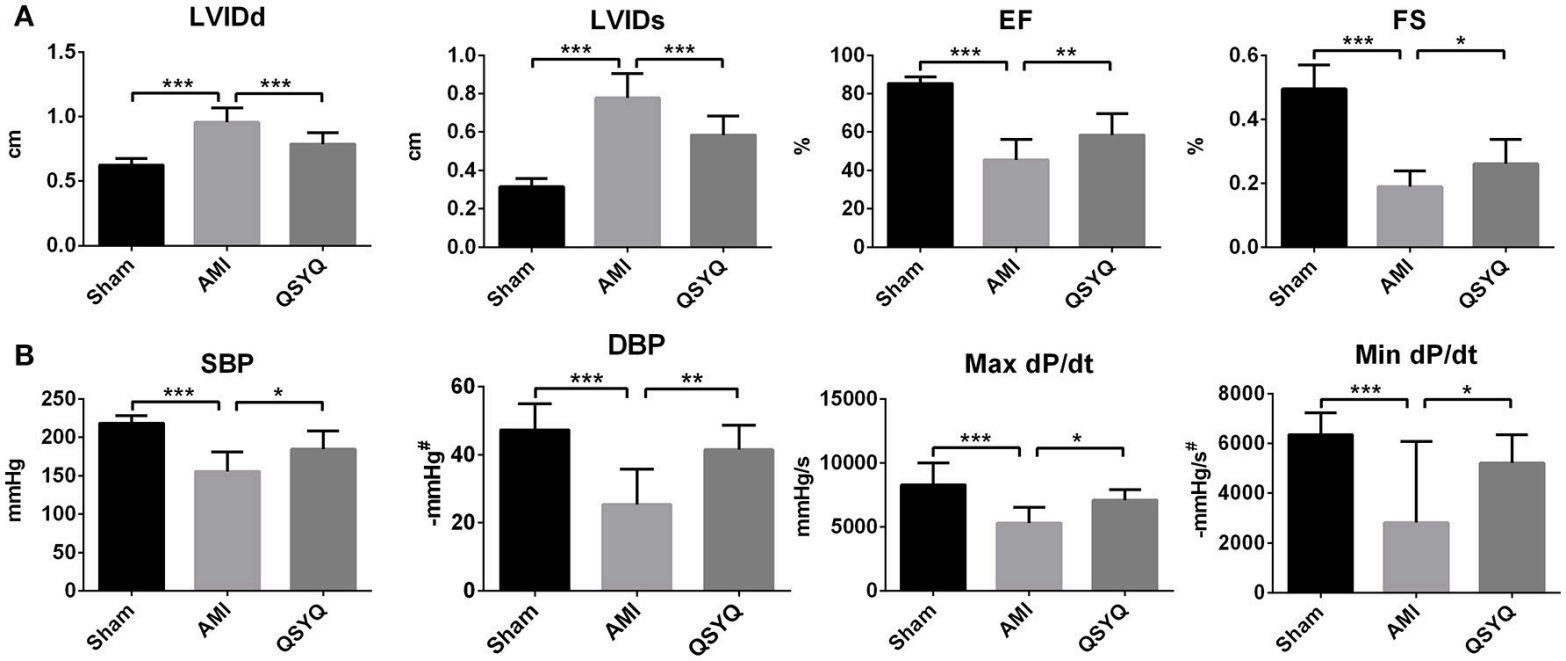
**The assessments of cardiac function by echocardiography and hemodynamics. (A)** Echocardiographic analysis was performed in Sham, AMI, and QSYQ (2.33 g/kg) groups for 28 days after LAD, *n* = 8 per group. **(B)** Hemodynamics analysis was carried out in each group. Data shown are the mean ± *SD, n* = 9 per group. ^*^P < 0.05; ^**^P < 0.01; ^***^P < 0.001. ^#^DBP and Min dP/dt are negative values.

Twenty-eight days after surgery, echocardiography showed down-regulation of EF and FS in AMI model group, with 46.68 and 61.67% decrease respectively as compared with Sham-operated group. This was accompanied by increase in LVIDd and LVIDs (53.14 and 147.49% increments, respectively), which suggested cardiac hypertrophy in AMI development. After treatment with QSYQ, EF, and FS were recovered by 28.53 and 37.20%, respectively. LVIDd and LVIDs of QSYQ-treated group were recovered by 17.55 and 24.85%, respectively (Figure 1A and Figure S2).

As shown in Figure 1B, in AMI model group, 2 indicators reflecting LV systolic function—systolic blood pressure (SBP) and Max dP/dt were reduced by 25.47 and 36.02% respectively, compared to Sham-operated group. In QSYQ-treated group, these indicators were significantly improved (5.8 and 33.61% increments, respectively). Diastolic blood pressure (DBP) and Min dP/dt, which were associated with LV diastolic function, in AMI model group were decreased by 46.47 and 37.16% respectively, compared to Sham-operated group. QSYQ treatment enhanced DBP and Min dP/dt by 63.78 and 30.41% respectively. No difference was observed in heart rate among 3 groups. Echocardiography and hemodynamic measurements demonstrated marked protective effects of QSYQ on improving cardiac function and hemodynamics.

### Global therapeutic effects of QSYQ on AMI and identification of keystone QSYQ-regulated genes

By comparison of AMI model with Sham-operated group, 868 DEGs were identified and regarded as AMI-pathogenic genes, including 418 up-regulated and 450 down-regulated genes (Figures S3A,D). The comparison of QSYQ-treated with AMI model group revealed 2,733 DEGs (Figures S3B,D), consisting of 1,344 up-regulated and 1,389 down-regulated genes. These 2,733 DEGs were considered as QSYQ-responsive genes. Out of these 2 comparisons, 500 genes overlapped and showed reverse differential expression, which were considered as QSYQ-regulated genes. These genes included 221 inversely up-regulated and 279 inversely down-regulated genes under QSYQ treatment (Figures S3C,D). As shown in Figure S3C, QSYQ exerted a significant modulation on global gene expression of AMI toward the normal expression level, which accounting for therapeutic effects of QSYQ on AMI. GCN was constructed with expression profile of 500 QSYQ-regulated genes in 3 groups of samples. PageRank (Brin and Page, 2012) further characterized the centrality of genes in GCN. Targeted network attack analysis (Figure S4) showed that the top 421 genes in PageRank ranking were the hub genes of GCN. Thus, they were considered as the “keystone QSYQ-regulated genes.”

A list of top 20 keystone QSYQ-regulated genes and their biological functions are given in Table S1. It is intriguing to note that most genes are related to extracellular matrix (ECM). ECM abnormality is well known to be associated with VR, which supports well relevance of our method for selection of keystone QSYQ-regulated genes. Expression of these genes was significantly up-regulated in AMI model compared with Sham-operated group. Furthermore, their increased expression was significantly reversed by QSYQ treatment.

Keystone QSYQ-regulated genes were mainly enriched in cardiovascular diseases and cardiac signaling pathways, including dilated cardiomyopathy, hypertrophic cardiomyopathy and β-adrenergic receptor (β-AR)-mediated signaling pathway (Figure S5).

### Significance analysis of functional group based on keystone QSYQ-regulated genes

**Figure 2:**
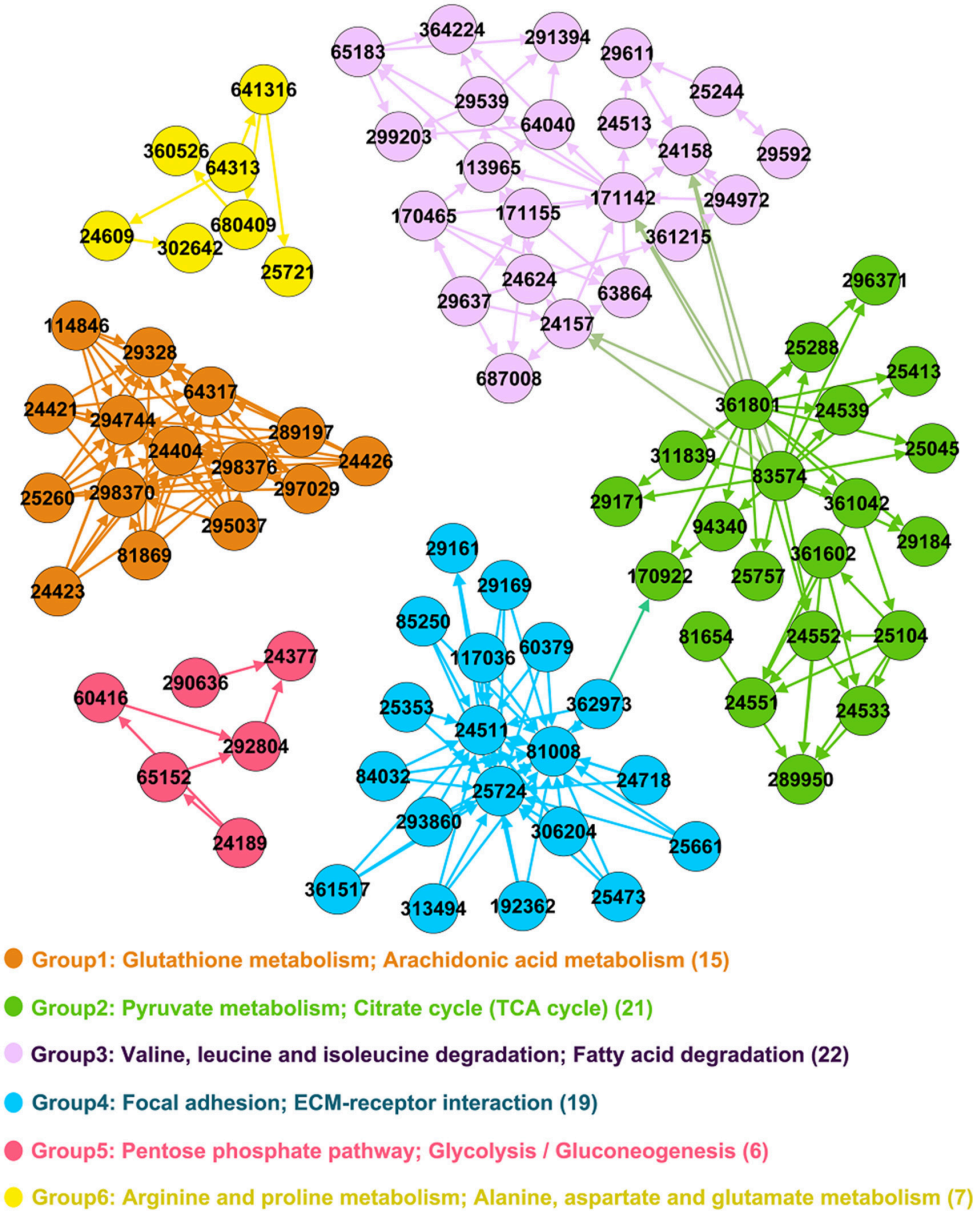
**Significant functional groups**. Genes are represented as nodes in the network and Entrez IDs are marked in the circle. The legend text denotes main KEGG pathways and the number in bracket denotes count of genes in functional group.

**Figure 3:**
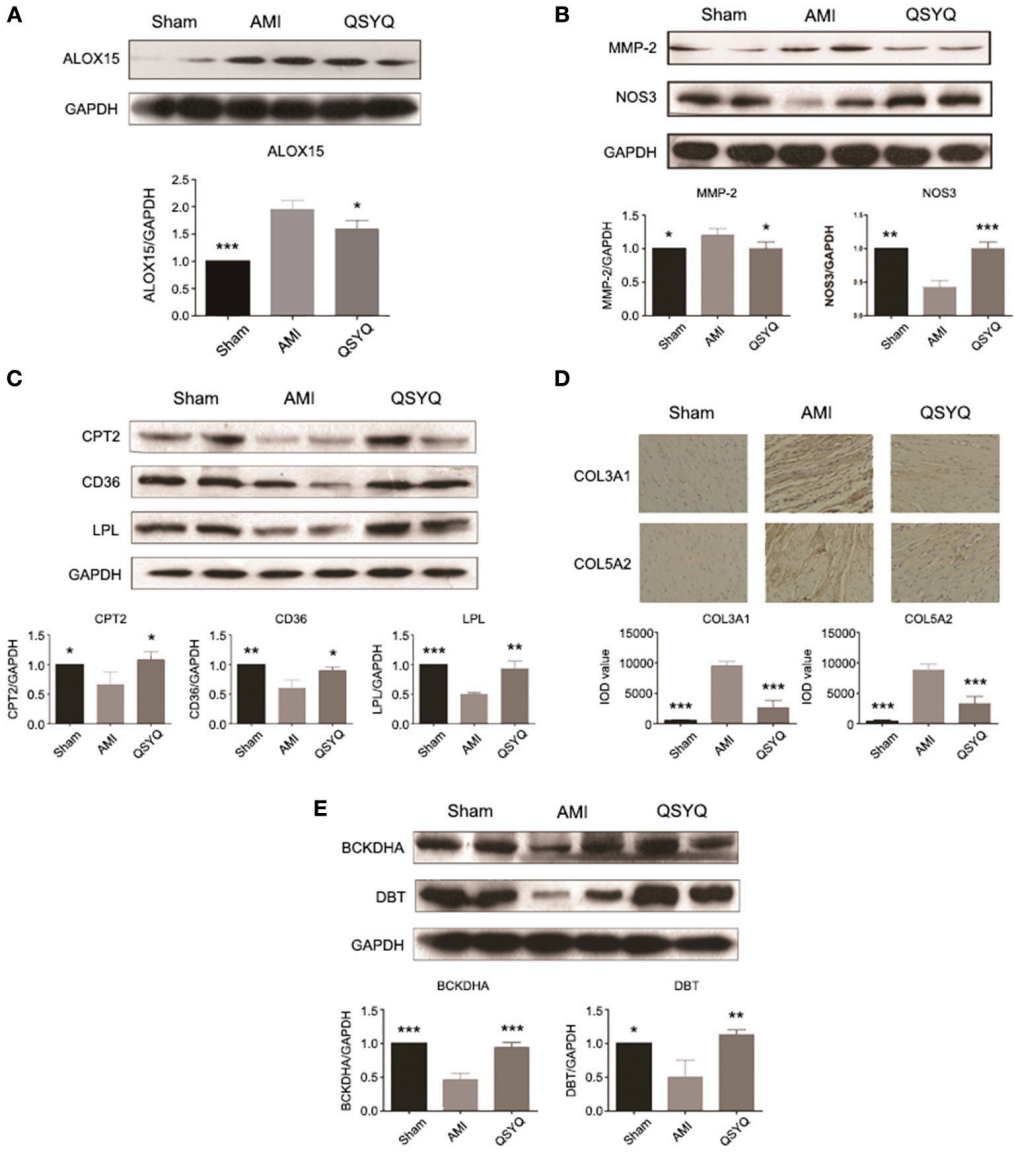
**Experimental validations of key genes of significant functional groups**. Data shown are the mean ± *SD*, ^*^P < 0.05; ^**^P < 0.01; ^***^P < 0.001. **(A)** QSYQ decreased gene expression level of ALOX15. *n* =3 per group. **(B)** QSYQ decreased gene expression level of MMP-2 and increased that of NOS3. *n* = 3 per group. **(C)** QSYQ increased cardiac CPT2, CD36 and LPL in rats with AMI. *n* = 3 per group. **(D)** QSYQ inhibited cardiac-generated collagens such as COL3A1 and COL5A2. *n* = 6 per group. **(E)** QSYQ increased cardiac BCKDHA and DBT in rats with AMI. *n* = 3 per group.

Significance of functional groups was evaluated by our new strategy based on priorities of keystone QSYQ-regulated genes (Materials and Methods). Table S2 demonstrates significance analysis for 15 functional groups. Significant functional groups were identified with adjusted *P* < 0.005 (Group 1–6 in Table 1 and Figure 2). Some QSYQ-responsive genes (not belong to functional groups), which participate main KEGG pathways of significant functional groups, were also included into analysis to help understand therapeutic effects of QSYQ. Key genes of functional groups and indicators of plasma lipid and lipoprotein levels were validated and showed in Table 2, Table S3, and Figure 3. Results of significant functional group analysis were shown as follows.

**Table 1:** **Significant functional groups**.

Number of groups^*^	**Enrichment score (ES)**	***P*-value**	**Adjusted *P*-value**	**Involved KEGG pathways**
Group 1	4.162	<0.0001	<0.0001	Glutathione metabolism, Arachidonic acid metabolism
Group 2	3.528	<0.0001	<0.0001	Pyruvate metabolism, Citrate cycle (TCA cycle)
Group 3	3.093	<0.0001	<0.0001	Valine, leucine and isoleucine degradation, Fatty acid degradation
Group 4	2.886	<0.0001	<0.0001	Focal adhesion, ECM-receptor interaction
Group 5	2.397	<0.0001	<0.0001	Pentose phosphate pathway, Glycolysis/Gluconeogenesis
Group 6	1.601	0.0002	0.0020	Arginine and proline metabolism, Alanine, aspartate and glutamate metabolism

* *Significant functional groups are ordered by ES decreasingly*.

**Table 2:** **Indicators of plasma lipid and lipoprotein levels in different groups**.

**Group**	**TC(mmol/L)**	**TG(mmol/L)**	**HDL(mmol/L)**	**LDL(mmol/L)**
Sham	0.75 ± 0.136^**^	0.064 ± 0.023^**^	0.54 ± 0.191^*^	0.03 ± 0.010^**^
AMI	1.30 ± 0.355	0.26 ± 0.060	0.34 ± 0.076	0.064 ± 0.019
QSYQ	0.84 ± 0.057^**^	0.14 ± 0.059^**^	0.44 ± 0.086	0.01 ± 0.006^**^

*Compared with AMI group*,

* P < 0.05;

** *P < 0.01. n = 6 per group*.

### QSYQ suppressed inflammatory response induced by arachidonic acid LOX pathway (Group 1)

Inflammatory response induced by arachidonic acid lipoxygenase (LOX) pathway was activated in AMI and markedly suppressed by QSYQ treatment (Figure S6). Expression of important genes in arachidonic acid LOX pathway were significantly up-regulated in AMI process, including arachidonate 15-lipoxygenase (ALOX15) and matrix metallopeptidase 2 (MMP-2) (Table S3). After QSYQ treatment, their expression was significantly decreased. Western blot of ALOX15 and MMP-2 (Figures 3A,B) further confirmed the regulatory effects of QSYQ on suppression of arachidonic acid LOX pathway in AMI.

### QSYQ enhanced NOS3 expression and elevated NO production (Group 6)

Production of nitric oxide (NO) from arginine metabolism catalyzed by nitric oxide synthase 3 (NOS3) was markedly increased after QSYQ treatment compared with AMI model ones (Table S3), which was also observed by Western blot (Figure 3B). Western blot also showed decreased expression of NOS3 in AMI model compared with Sham-operated group.

### QSYQ ameliorated dyslipidaemia via up-regulating fatty acid oxidation (Group 3)

Blood biochemical analyses were performed to evaluate plasma lipid levels. Compared with Sham-operated, levels of TC, TG, and LDL in AMI model group increased by 72.76, 306.25, and 140.00%, respectively. HDL level decreased by 35.70% in AMI model compared with Sham-operated group. After treatment with QSYQ, levels of TC, TG and LDL decreased by 35.46, 46.15, and 84.37% respectively, as compared with model group. HDL level was up-regulated by 28.49%. These findings suggested that QSYQ ameliorated dyslipidaemia in rats with AMI (Table 2).

As an important pathway to remove lipids from heart, fatty acids oxidation was significantly inhibited in AMI and reversely up-regulated after QSYQ treatment (Figure S7). The affected genes included lipoprotein lipase (LPL), cluster of differentiation 36 (CD36), acyl-CoA synthetase long-chain family member 1 (ACSL1) and carnitine palmitoyltransferase 2 (CPT2) (Table S3). Western blot confirmed significant reverse expression changes of 3 key enzyme genes: LPL, CD36, and CPT2 (Figure 3C).

### QSYQ inhibited cardiac VR (top keystone QSYQ-regulated genes and Group 4)

A large proportion of top keystone QSYQ-regulated genes are related with ECM. Of note, abnormal elevated expression of these ECM-related genes was effectively inhibited by QSYQ treatment. These ECM-related genes include transforming growth factor beta 1 induced transcript 1 (TGFB1I1) and lipopolysaccharide-induced TNF factor (LITAF) (Table S3). ECM abnormality is regarded as a hallmark of VR. Moreover, in functional group 4, it was also revealed inverse expression alterations in ECM genes induced by QSYQ treatment (Figure S8), including collagen type III alpha 1 (COL3A1) and collagen type V alpha 2 (COL5A2) (Table S3). IHC was applied to confirm the inverse alterations of COL3A1 and COL5A2 (Figure 3D), which supported the regulatory effects of QSYQ to ameliorate VR in AMI.

### QSYQ accelerated BCAAs degradation and other energy metabolism (analyses of Group 2, 3, and 5)

Up-regulation of branched-chain amino acids (BCAAs) degradation by QSYQ treatment was identified in our study (Figure S9). BCAA homeostasis is controlled by mitochondrial branched-chain alpha-keto acid dehydrogenase complex (BCKDC). 4 genes (BCKDHA, BCKDHB, DBT, and DLD) encoding the catalytic subunits of BCKDC, were suppressed in AMI model rats compared with Sham-operated ones (Table S3). After QSYQ treatment, 2 of them were significantly up-regulated: BCKDHA and DBT, which were confirmed by Western blot (Figure 3E). The regulatory effects of QSYQ on BCAAs degradation may mediate cardiovascular contractility and compliance (Details in figure notes of Figure S9). In addition, other energy metabolism including glycolysis (PRKAB1 and ALDOA), TCA cycle (SUCLG1 and SUCLA2) and creatine metabolism (CKM and CrT) were also accelerated by the treatment of QSYQ (Table S3).

## Discussion

### Therapeutic effects of QSYQ: anti-inflammation, ameliorate dyslipidemia, and VR progression

#### QSYQ attenuates inflammation through suppressing arachidonic acid LOX pathway and elevating production of NO

Inflammation mediated by arachidonic acid (AA) metabolism pathway plays a critical role in AMI progression (Levick et al., 2007; Wang et al., 2014). Our study identified significant reverse gene expression changes in AA metabolism, especially in arachidonic acid LOX pathway (Figure S6). AA is a kind of polyunsaturated fatty acids, which is metabolized by 3 types of enzymes: lipoxygenase (LOX), cyclooxygenase (COX), and cytochrome P-450 (CYP) (Funk, 2001; Moreno, 2009). As shown in Figure S6, in the LOX-catalyzed sub-pathway, ALOX15 and ALOX5 can form hydroperoxyeicosatetraenoic acids (HPETEs), which are further converted to hydroxyeicosatetraenoic acids (HETEs) by glutathione peroxidases. Previous studies have shown that hypoxia increases the proinflammatory enzyme ALOX15 in human carotid plaques (Magnusson et al., 2012). Furthermore, ALOX15 induces production of the reactive signaling molecule 15-HPETE, which is a peroxidised lipid. Of note, increased levels of peroxidised lipids are tightly connected with complex inflammatory diseases such as AMI (Spiteller, 2006). ALOX15 also catalyzes production of HETE. HETE enhances the adhesion of leukocytes to endothelium by activating chemokine production, which is an early event in development of AMI (Hedrick et al., 1999). Ye et al. (2004) has reported that 5-HETE induced the expression of MMP-2, which supports our results. It is noted that matrix metalloproteinases (MMPs) are involved in the degradation of matrix components and contribute to VR progression in AMI. Thus, our results demonstrated that the enhanced expression levels of ALOX15 and ALOX5, contribute to accumulation of pro-inflammation effects by HPETEs and HETEs, and further exacerbate VR progression induced by MMPs. On the other hand, increased expression of ALOX5AP accelerates the accumulation of LTA4 and thus enhances AMI. Interestingly, QSYQ treatment effectively suppresses inflammation by down-regulating the key genes including ALOX15 and ALOX5AP in arachidonic acid LOX pathway, and finally inhibits VR indicators, including MMP-2 and MMP-23.

Meanwhile, NO synthesized from L-arginine by NOS3 can reduce inflammation and oxidative stress through inhibiting lipid peroxidation and scavenging superoxide anion (Katakami et al., 2014), and thus alleviates VR in AMI progression.

#### QSYQ ameliorates dyslipidaemia through elevating CD36-CPT2-LPL fatty acid oxidation

Dyslipidaemia is closely associated with exacerbation of AMI. Our study demonstrated that QSYQ ameliorates the dyslipidaemia through elevating CD36-CPT2-LPL fatty acid oxidation.

The initial step of fatty acid metabolism is hydrolysis of TG. LPL is the major enzyme responsible for hydrolysis of TG. Moreover, LPL activity is positively correlated with HDL levels (Blades et al., 1993; Tornvall et al., 1995). As an AMI Risk in Communities Study shown, increased AMI risk has strong associations with TC, LDL-C, and TG; while decreased AMI risk is closely related with HDL-C (Sharrett et al., 2001). It was demonstrated in the study that QSYQ decreases plasma TC, TG, and LDL levels and up-regulate LPL expression and plasma HDL levels, which effectively ameliorated dyslipidaemia and decreased AMI risk.

After hydrolysis of TG into fatty acids, the next step is the transportation of fatty acids from extracellular environment to cytoplasm. CD36 as a fatty acid translocase, binds and transports long chain fatty acids. Previous study (Krzystolik et al., 2015) has suggested protective effect of higher soluble CD36 in coronary artery disease patients. Meanwhile higher soluble CD36 concentration is also associated with lower risk of LV hypertrophy (Krzystolik et al., 2015). Thus, overexpression of CD36 induced by QSYQ is associated with lower risk of LV hypertrophy.

Once transported into cytoplasm, fatty acids participate in β-oxidation in mitochondria. It is noted that ACSL1, CPT2, and ACADM genes, which encode key enzymes of fatty acid β-oxidation, were significantly suppressed in AMI progression and reversely enhanced after QSYQ treatment. ACSL1 is highly expressed in oxidative tissues like brown adipose tissue and heart (Ellis et al., 2011). Activated fatty acid normally provide 60–90% of the substrate used by the heart for energy, but when ACSL1 is absent, fatty acid oxidation decreases more than 90% (Ellis et al., 2011). CPT2 together with carnitine palmitoyltransferase 1 (CPT1), controls the rate of long-chain acyl-CoA transporting into mitochondria from cytoplasm.

#### QSYQ alleviates VR progression

VR markers were elevated in AMI progression. QSYQ treatment suppressed expression of VR markers as our results shown. In atherosclerotic lesions, levels of ECM components, particularly fibrillar collagen such as COL3A1 and COL5A2, are elevated (Tyagi, 1999), which were validated in our study. Furthermore, we identified main causes of VR, inflammation and dyslipidaemia were both ameliorated by QSYQ treatment.

### Overview of multipronged therapeutic effects of QSYQ on AMI

**Figure 4:**
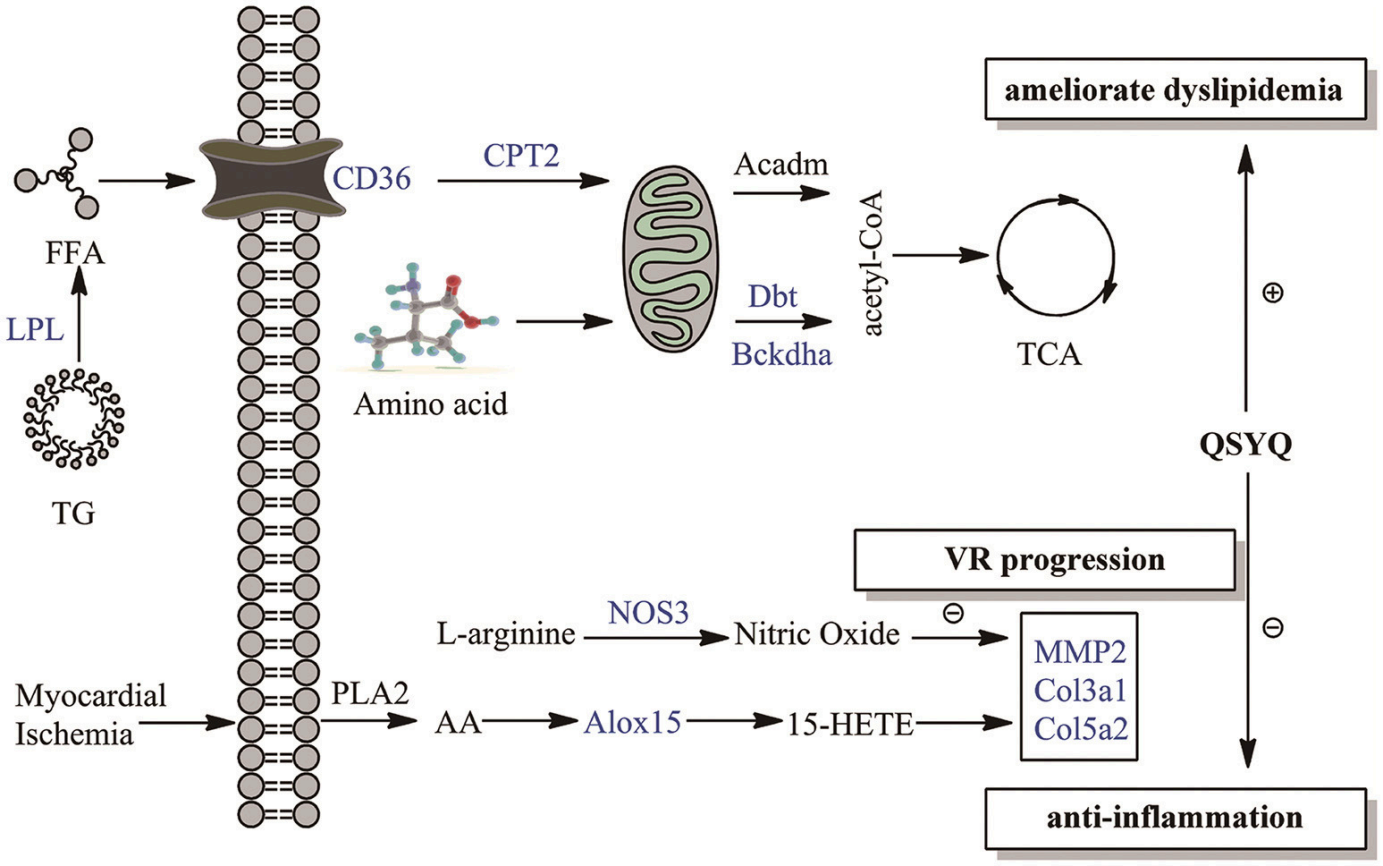
**Overview of multipronged therapeutic effects of QSYQ on AMI**. Therapeutic effects of QSYQ are highlighted with blue in our study: anti-inflammation, ameliorate dyslipidemia and VR progression. Molecules in the figure were drawn with ChemBioDraw Ultra 12.0.

Multipronged therapeutic effects of QSYQ were summarized in Figure 4 and Table S4. VR caused by inflammation and dyslipidaemia is considered as a progressive yet irreversible process and the most major pathological manifestation of AMI (Sharrett et al., 2001; Tabas and Glass, 2013). In present study, VR markers, such as COL3A1, COL5A2, and MMP-2, were elevated in AMI progression. QSYQ treatment alleviated VR through counter-acting the aforementioned events. Furthermore, novel therapeutic effects of QSYQ were proposed in our study. QSYQ can inhibit inflammatory response by down-regulating arachidonic acid LOX pathway and elevating production of NO. Meanwhile, QSYQ can ameliorate dyslipidaemia through elevating CD36-CPT2-LPL fatty acid oxidation. Improvement of fatty acid metabolism effectively increases energy supply to cardiac contractility and relaxation in AMI and thus evaluates EF value. In summary, QSYQ concurrently alleviated VR progression, attenuated inflammation induced by arachidonic acid LOX pathway and NO production, and ameliorated dyslipidaemia in the treatment of AMI, thus improved cardiac function and hemodynamics. TCM has upheld the holistic therapeutic philosophy for more than 2000 years. Many Chinese herbal formulae are multi-targeting in the treatment of diseases. Our observations here support a multi-targeting mechanism for QSYQ in the treatment of AMI.

### New strategy–keystone gene-based group significance analysis

Many traditional strategies for network group significance analysis focus on topological properties. For instance, MCODE algorithm evaluates group significance with a cluster score, to emphasize densely connected clusters (groups). However, density cannot reflect the degree to which the group is relevant with regulatory effects of QSYQ in our study. Our strategy introduces quantitative information (PageRank scores of keystone QSYQ-regulated genes) to character the regulatory effects of QSYQ when analysing the significance of functional group. To some extent, our strategy is a particular enrichment test method which is analogous to Gene Set Enrichment Analysis (Subramanian et al., 2005).

Most enrichment test methods adopt overlap statistics (Berriz et al., 2003; Doniger et al., 2003; Zhong et al., 2004; Subramanian et al., 2005), which only concern the count of overlap between the important genes and genes of a certain pathway or ontology term. However, they do not take advantage of the priorities of important genes and give equal weight to every gene. Compared with these enrichment test methods, our strategy differs in two regards. First, it underscores both the priorities and count of keystone genes in functional group. When genes of a group mainly distribute on the top of priority list of keystone genes, the term *P*_*hit*_ is larger and then ES is larger. Meanwhile, if there are more keystone genes in the group, corresponding *P*_*miss*_ is smaller and then ES is larger. Second, our strategy assesses the significance level of ES by Monte Carlo simulation that sample genes from the background network. This process preserves the enrichment degree of observed functional group with QSYQ-responsive genes, and thus, provides an accurate null distribution. Keystone gene-based group significance analysis has a broad application in evaluation of group significance with priorities of genes.

## Conclusions

Based on comprehensive transcriptome analyses and experimental validation, we identified therapeutic effects of QSYQ on AMI at multiple biological levels, including gene expression, pathways involved and functional group. We concluded that QSYQ concurrently alleviated VR progression, attenuated inflammation induced by arachidonic acid LOX pathway and NO production, and ameliorated dyslipidaemia in the treatment of AMI. Moreover, our study provided a new strategy to analyse the significance of functional group based on the priorities of keystone genes, which has a broad application in pharmacological studies.

## Author contributions

Each author has contributed significantly to the submitted work. WW and RZ conceived and designed the experiments. YW, WL, and CL performed the experiments. YW, WL, CL, SS, GJ, LZ, and LL analyzed the data. YW, WL, CL, SS, GJ, LZ, LL, RZ, and WW wrote the paper. All authors read and approved the final manuscript.

### Conflict of interest statement

The reviewer JL declared a shared affiliation, though no other collaboration, with several of the authors YW, CL, LL, and WW to the handling Editor, who ensured that the process nevertheless met the standards of a fair and objective review. The other authors declare that the research was conducted in the absence of any commercial or financial relationships that could be construed as a potential conflict of interest.

## Abbreviations

AMI: Acute myocardial infarction
HF: Heart failure
TCM: Traditional Chinese medicine
QSYQ: Qishenyiqi
IHC: Immunohistochemistry
SD: Sprague-Dawley
LVIDd: Left ventricular internal diameter at end-diastole
LVIDs: Left ventricular internal diameter at end-systole
EF: Ejection fraction
FS: Fractional shortening
LV: Left ventricular
GCN: Gene co-expression network
FDR: False discovery rate
ES: Enrichment score
TC: Total cholesterol
TG: Triglyceride
HDL: High density lipoprotein
LDL: Low density lipoprotein
ECM: Extracellular matrix
AA: Arachidonic acid
LOX: Lipoxygenase
ALOX15: Arachidonate 15-lipoxygenase
ALOX5: Arachidonate 5-lipoxygenase
15-HPETE: 15-Hydroxyeicosatetraenoic acid
MMP: Matrix metalloproteinase
MMP-2: Matrix metallopeptidase 2
MMP-23: Matrix metallopeptidase 23
VR: Ventricular remodeling
ALOX5AP: Arachidonate 5-lipoxygenase activating protein
NO: Nitric oxide
NOS3: Nitric oxide synthase 3
CD36: Cluster of differentiation 36
ACSL1: Acyl-CoA synthetase long-chain family member 1
CPT2: Carnitine palmitoyltransferase 2
CPT1: Carnitine palmitoyltransferase 1
LPL: Lipoprotein lipase
ACADM: Acyl-CoA dehydrogenase, C-4 to C-12 straight chain
BCKDHA: Branched chain ketoacid dehydrogenase E1, alpha polypeptide
DBT: Dihydrolipoamide branched chain transacylase E2
COL3A1: Collagen, type III, alpha 1
COL5A2: Collagen, type V, alpha 2
HPETE: Hydroperoxyeicosatetraenoic acid
HETE: Hydroxyeicosatetraenoic acid.
