# Supplementary material for: Multipronged Therapeutic Effects of Chinese Herbal Medicine Qishenyiqi in the Treatment of Acute Myocardial Infarction
Source: Front Pharmacol. 2017 Mar 2;8:98. doi: 10.3389/fphar.2017.00098 (PMC5332380; doi:10.3389/fphar.2017.00098)
Supplement: Supplementary file 1 [file DataSheet1.DOC]

***Supplemental material***

**Multipronged Therapeutic Effects of Chinese Herbal Medicine Qishenyiqi in the Treatment of** **Acute Myocardial Infarction**

Yong Wang^1,#^, MD; Weili Lin^2,#^, PhD; Chun Li, MD^3^; Sarita Singhal^4^, MD; Gaurav Jain^5^, MD; Lixin Zhu^4,6,7^, PhD; Linghui Lu^1^, MD; Ruixin Zhu^8^,*, PhD; Wei Wang^1^,*, MD

^1^ Basic Medical College, Beijing University of Chinese Medicine, Beijing 100029, P.R.China

^2^ Key Lab of Computational Biology, CAS-MPG Partner Institute for Computational Biology, Shanghai Institutes for Biological Sciences, Chinese Academy of Sciences, Shanghai 200031, P.R. China

^3^ Modern Research Center for Traditional Chinese Medicine, Beijing University of Chinese Medicine, Beijing 100029, P.R.China

^4^ Digestive Diseases and Nutrition Center, Department of Pediatrics, The State University of New York at Buffalo, Buffalo, New York 14214, United States.

^5^ Niagara Falls memorial medical center and Invision health, Buffalo, New York 14302, United States.

^6^ Genome, Environment and Microbiome Community of Excellence, The State University of New York at Buffalo, Buffalo, New York 14214, United States.

^7^ Institute of Digestive Diseases, Longhua Hospital, Shanghai University of Traditional Chinese Medicine, Shanghai 200032, P.R. China

^8^ Department of Bioinformatics, School of Life Sciences and Technology, Tongji University, Shanghai 200092, P.R.China.

^#^ Contributed equally

**Address for correspondence**

Ruixin Zhu ([rxzhu@tongji.edu.cn](mailto:rxzhu@tongji.edu.cn))

Department of Bioinformatics, School of Life Sciences and Technology, Tongji University, 1239 Siping Road, Shanghai 200092, P.R.China.

Tel: 86-21-6598-0296, Fax: 86-21-6598-1041

And

Wei Wang, wangwei@bucm.edu.cn

Basic Medical College, Beijing University of Chinese Medicine, Bei San Huan Dong Lu 11, Chao Yang district, Beijing 100029, P.R.China

Tel: 86-10-6428-6508, Fax: 86-10-6428-7545

**Figure S1: HPLC-DAD-MS^n^ analysis of QSYQ.**

A


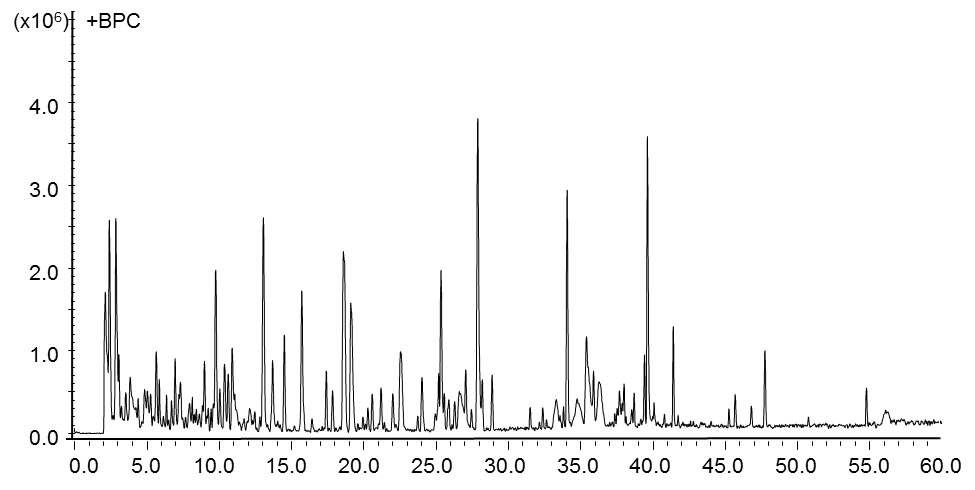

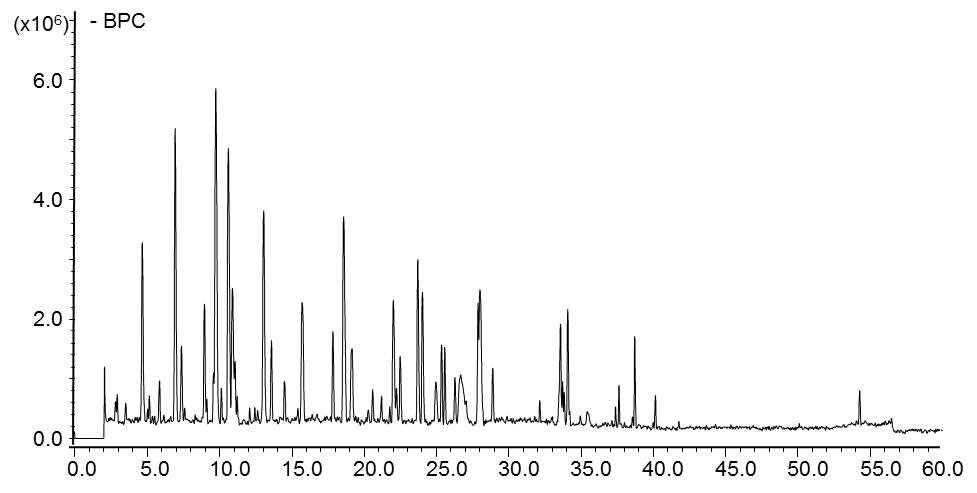


B

Figure S1. HPLC-DAD-MS^n^ analysis of QSYQ. (A) The ESI-MS/MS total ion current chromatogram of QSYQ in positive mode. (B) The ESI-MS/MS total ion current chromatogram of QSYQ negative mode.

We got QSYQ fingerprint by applying the HPLC-IT-TOF methods. Briefly, QSYQ comprises 6 medical herbs, namely *Astragalus membranaceus* (Fisch.) Bunge (‘huang-qi’ in Chinese), *Salvia Miltiorrhiza* Bunge (‘dan-shen’ in Chinese), *Lonicera japonica* Thunb., *Scrophularia aestivalis* Griseb., *Aconitum fischeri* Rchb., and *Glycyrrhiza uralensis* Fisch..

The procedure and results were list as supplement data (Figure S1) as follows:

MS-grade acetonitrile is product of Fisher Scientific (FairLawn, NJ, USA). Formic acid is product of Sigma Aldrich (St. Louis, MO, USA). Ultrapure water was prepared in our own laboratory by Milli-Q plus System (Millipore, Bedford, MA, USA). Analytical-grade solvents used for sample preparation are products of Beijing Chemical Factory (Beijing, China).

**Sample preparation for analysis**

QSYQ was weighed accurately (0.2 g) and placed into a 25 mL flask containing 10 mL 3% acetonitrile. Ultrasonic extraction (ultrasonic cleaner, 100 W, Fisher Scientific, USA) was then performed at room temperature for 5 min. The resulting mixture was filtered through a 0.22 μm membrane.

**HPLC-IT-TOF-MS^n^ conditions for chemical profile**

The HPLC-MS analysis was carried out on a Shimadzu HPLC-IT-TOF mass spectrometer equipped with electrospray ionization (ESI) source. The chromatographic separation was performed on a Zorbax SB C_18_ column (250 × 4.6 mm, 5 μm, Agilent) at 35 ºC. Acetonitrile (A)–0.04% formic acid (B) were used as the mobile phase for analysis. The flow rate was set at 1.0 mL/min. The elution condition was applied with a gradient program as follows: 0–10 min, 3-10% A; 10–12 min, 10–15% A; 12–35 min, 15–35% A; 35–40 min, 35–40% A; 40–50 min, 40–60% A. The optimized MS operating conditions were as follows: positive and negative mode; nebulizer gas (N_2_) flow, 1.5 L/min; CDL temperature, 200 ºC; heat block temperature, 200 ºC; detector voltage, 1.60 kV; interface voltage (+), 4.5 kV; Ion accumulated time, 10 ms; repeat times, 1; collision energy was set at 50% both for MS^2^, and MS^3^; pressure of ion trap, 1.9e-002 Pa; pressure of TOF region, 1.2e-004 Pa; scan range m/z 100–1000; precursor ion isolation, 3.0000 Da. An automatic scan mode was used in the HPLC-ESI-MS^n^ analysis. The HPLC effluent was introduced into the ESI source in a post-column splitting ratio of 1:4. Aliquots of 10 μL were injected into HPLC-MS system for analysis. The typical chromatograms are shown in FigureS 1.

**Figure S2. Detection of cardiac functions by echocardiography.**


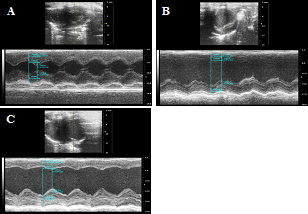


Figure S2. Detection of cardiac functions by echocardiography. (A) Normal cardiac function including LVEF and LVFS in Sham-operated group. (B) Down-regulation of LVEF and LVFS in AMI model group rats. (C) QSYQ can significantly up-regulate the echocardiography parameters especially EF and FS.

**Figure S3. Inverse gene expression changes induced by treatment of QSYQ.**


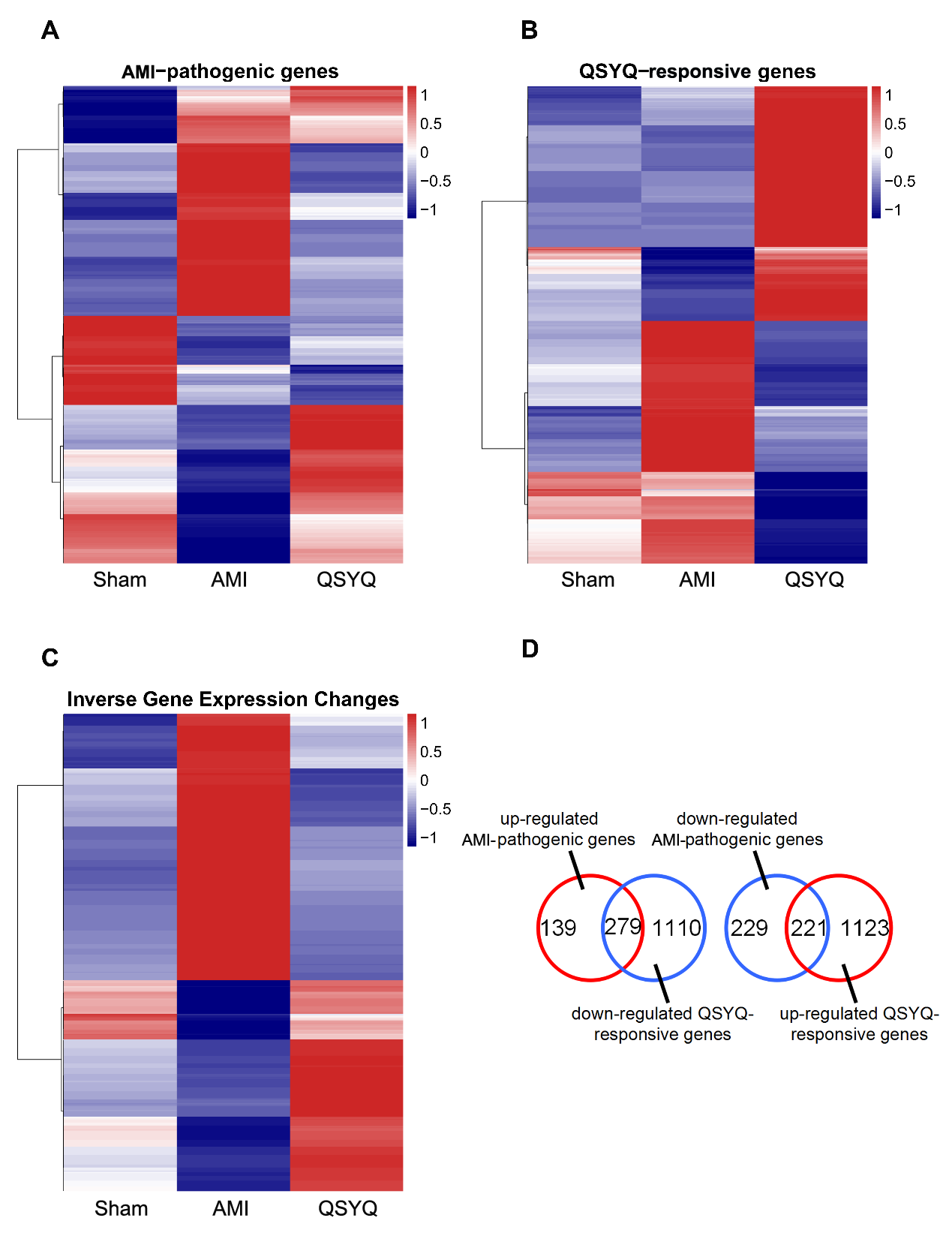


Figure S3. Inverse gene expression changes induced by treatment of QSYQ. (A) Heatmap of 868 DEGs in comparison of AMI with Sham group and these DEGs are regarded as AMI-pathogenic genes. (B) Heatmap of 2733 DEGs in comparison of QSYQ with AMI group and these DEGs are regarded as QSYQ-responsive genes. (C) Heatmap of 500 QSYQ-regulated genes, which are the overlap and show reverse differential expression between AMI-pathogenic genes and QSYQ-responsive genes. (D) Concrete distribution of QSYQ-regulated genes.

**Figure S4. Targeted attack on GCN.**


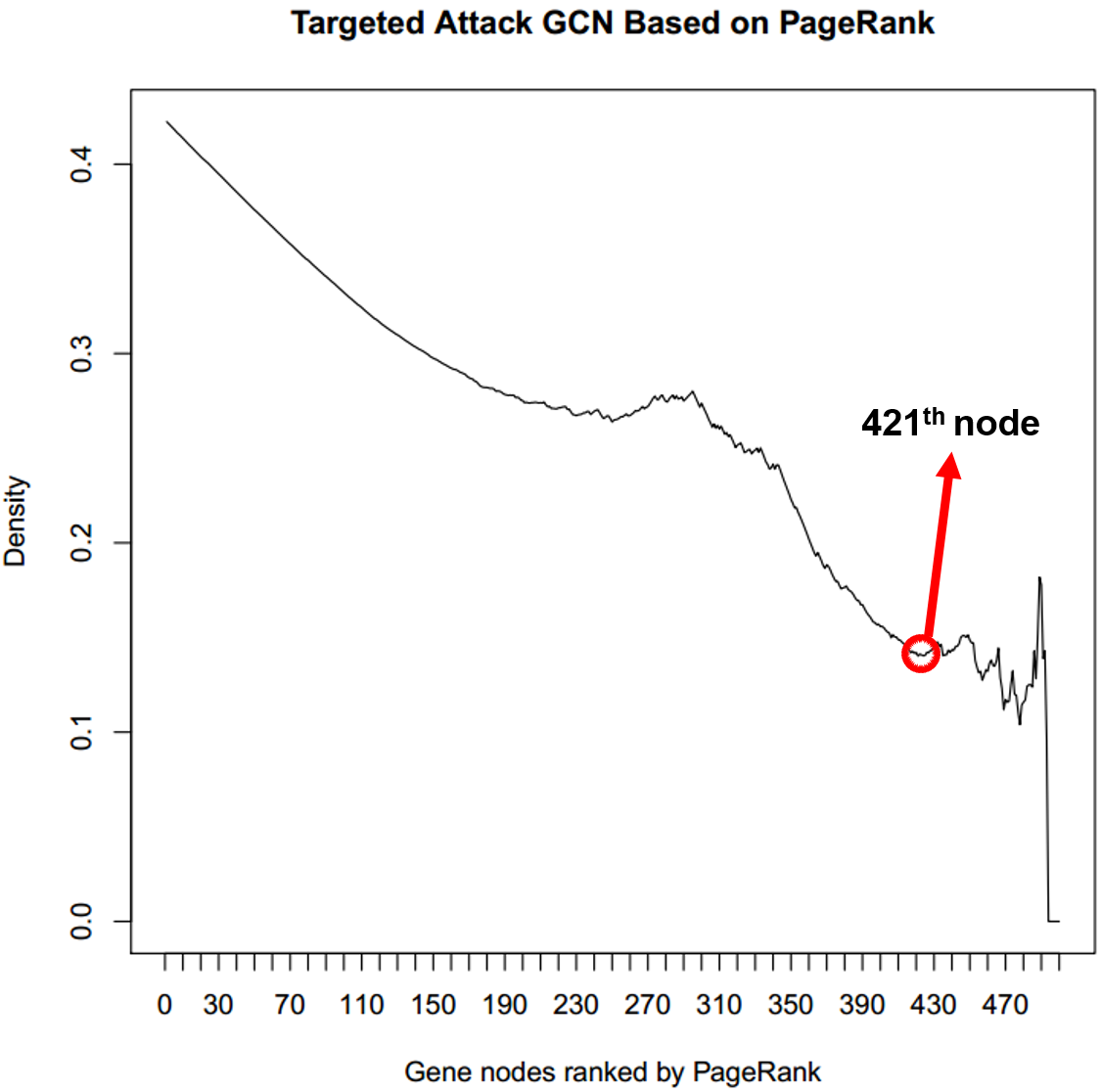


Figure S4. Targeted attack on GCN. The x-axis represents gene nodes which are sequentially removed from GCN in decreasing order of PageRank scores. The y-axis indicates the density of GCN. The curve depicts the destructive effect of targeted attack by sequentially removing the nodes in decreasing order of PageRank scores.

**Figure S5. KEGG pathway enrichment analysis of keystone QSYQ-regulated genes.**

**
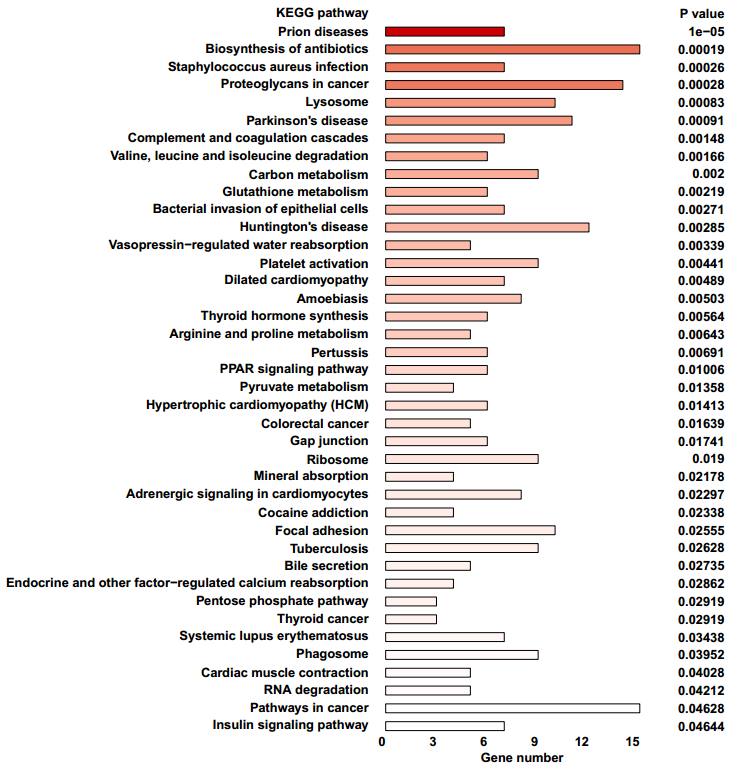
**

Figure S5. KEGG pathway enrichment analysis of keystone QSYQ-regulated genes. The number above the pillar in graph is the P value of enriched pathway. Darker red color indicates the smaller P value. The number of genes in the enriched pathway is represented as the height of pillar.

**Figure S6. Diagram of significantly reverse gene expression changes in arachidonic acid (AA) metabolism in AMI progression and treatment of QSYQ.**


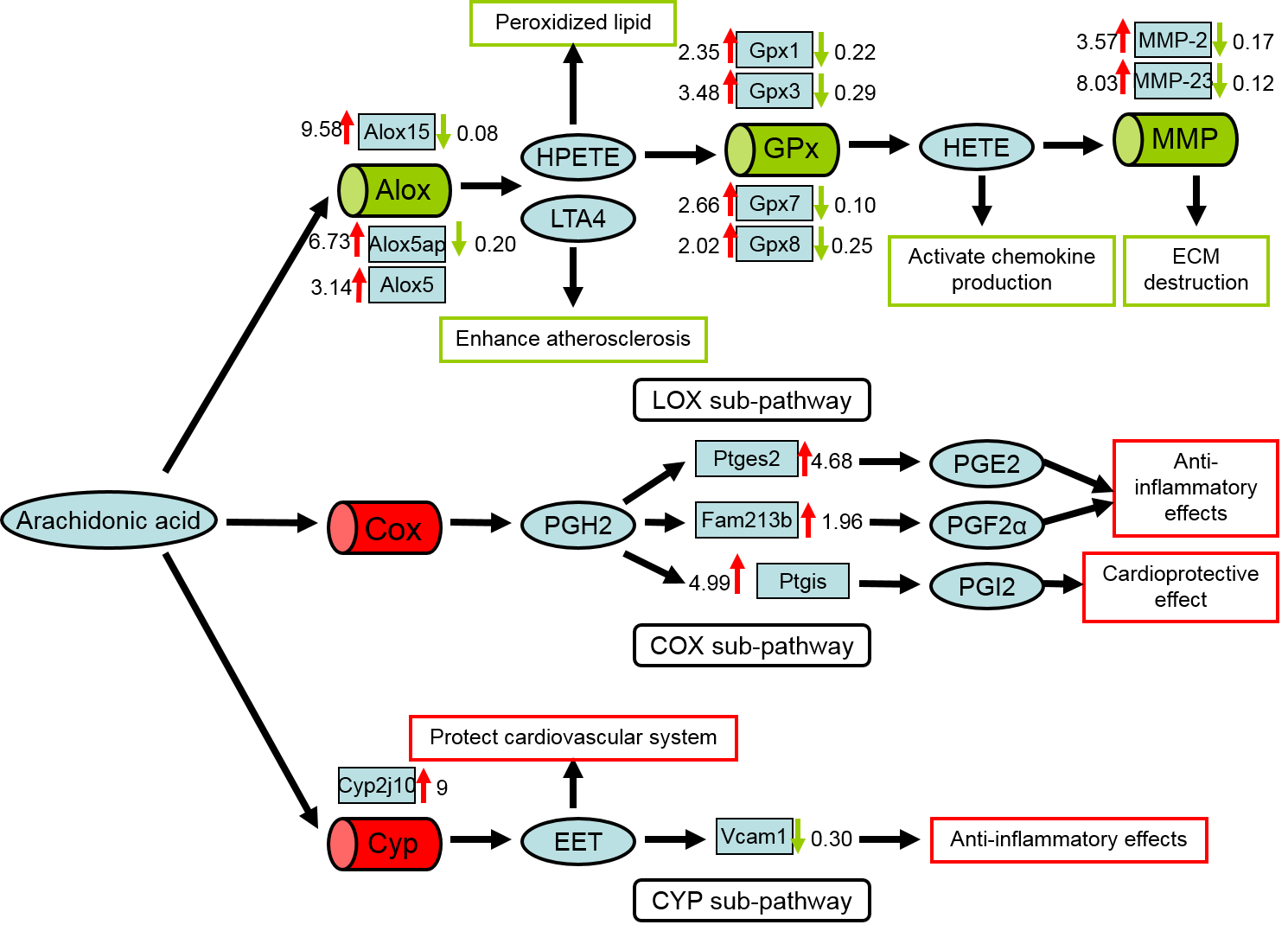


Figure S6. Diagram of significantly reverse gene expression changes in arachidonic acid (AA) metabolism in AMI progression and treatment of QSYQ. For one gene, left arrow and number indicate fold change for comparison of AMI model with Sham-operated group; right arrow and number represent fold change for comparison of QSYQ-treated with AMI model group.

Arachidonic acid (AA) is a kind of polyunsaturated fatty acids, which is released following activation of phospholipase A2s and subsequently metabolized by 3 types of enzymes: lipoxygenase (LOX), cyclooxygenase (COX) and cytochrome P-450 (CYP) . As shown in Figure S6, in the LOX-catalyzed sub-pathway, ALOX15 and ALOX5 can form hydroperoxyeicosatetraenoic acids (HPETEs), which are further converted to hydroxyeicosatetraenoic acids (HETEs) by glutathione peroxidases (including GPX1, GPX3, GPX7 and GPX8). Furthermore, COX and ALOX5 pathways catalyze AA to produce prostaglandins and leukotrienes. Both of them are targets of approved drugs . In our study, the increased expression of ALOX15 with 9.58 fold change, was observed in AMI model rats compared with Sham-operated ones. QSYQ could markedly down-regulate ALOX15 with 0.08 fold change. It has been reported that ALOX15 induces production of the reactive signaling molecule 15-HPETE, which is a peroxidized lipid that specifically phosphorylates threonine/serine kinases . Of note, increased levels of peroxidized lipids are tightly connected with complicated inflammatory diseases such as AMI . ALOX15 also catalyzes production of HETE. HETE enhances the adhesion of leukocytes to endothelium by activating chemokine production, which is an early event in development of AMI . Magnusson *et al.* observed that hypoxia increases the proinflammatory enzyme ALOX15 in human carotid plaques and a 17-fold up-regulation of ALOX15 mRNA expression in the ischemic heart biopsies from patients who underwent coronary bypass surgery, compared with non-ischemic cardiac tissue , which greatly supports to our results. In addition, Magnusson *et al.* have showed increased cathepsin B expression in the ischemic heart tissue compared with non-ischemic heart . Cathepsin B is reported to be released from malignant cells after simulation with the 15-lipoxygenase enzyme products, including ALOX15 . Interestingly, our results also found that 3 cysteine protease cathepsins (including cathepsin H, cathepsin L and cathepsin K) were increased in AMI progression and were down-regulated induced by QSYQ treatment. Thus, we could postulated that the increment of 3 cysteine protease cathepsins may be simulated with ALOX15, In addition, cysteine protease cathepsins show protease activity of degrading ECM remodeling related proteins and affect the immune and inflammatory actions.

Another LOX, expression of ALOX5 was slightly increased in comparison of AMI model with Sham-operated group, with 0.08 P value and 3.14 fold change. However, arachidonate 5-lipoxygenase activating protein (ALOX5AP) was found to be inversely down-regulated after QSYQ treatment. Like ALOX15, ALOX5AP was significantly up-regulated with 6.73 fold change in AMI progression but was strongly inhibited with 0.20 fold change under QSYQ treatment. ALOX5AP which localizes to the plasma member, translocates ALOX5 from the cytoplasm to the cell membrane and actives ALOX5. ALOX5AP with ALOX5 is required for leukotriene synthesis in AA metabolism. Hence, the increased activity of ALOX5AP enzyme leads to accumulation of leukotrienes LTA4, which is important inflammatory mediator and leads to enhanced AMI . The results about ALOX5AP in our study were also supported by previous studies. It has been demonstrated that the one genetic variant of ALOX5AP: rs4769874 significantly increased the risk of coronary artery disease (CAD), while another genetic variant of ALOX5AP: rs9579646 was associated with decreased CAD risk . The rs4769874 variant is most likely linked to the enhanced expression of ALOX5AP and its gene product 5-lipoxygenase activating protein (FLAP), hence amplifying the leukotriene pathway inflammatory reactions and atherogenic effects. On the contrary, the rs9579646 variant revealed a decreased expression of the ALOX5AP gene and its gene product FLAP, which leads to a significantly lower CAD risk in the study population and diminishes the drastic effects of the leukotriene pathway .

In LOX sub-pathway, expressions of several glutathione peroxidases including GPX1, GPX3, GPX7 and GPX8 were enhanced in AMI progression and be suppressed after QSYQ treatment. These enzymes are involved the conversion of 5(S)-HPETE and 15(S)-HPETE into 5(S)-HETE and 15(S)-HETE, respectively. Matrix metalloproteinases (MMP) are involved in the degradation of matrix components and Ye *et al*. has reported that 5-HETE induced the expression of MMP-2. More importantly, besides MMP-2, MMP-23 was also revealed highly expressed in AMI progression and was effectively suppressed after treatment of QSYQ.

Thus, we can make the hypothesis that the enhanced expression of ALOX15 and ALOX5 contributes to increased HPETEs, which are subsequently converted to HETEs by glutathione peroxidase. Moreover, highly expression of several glutathione peroxidases including GPX1, GPX3, GPX7 and GPX8, contributed to the increment of HETE. Some HETEs such as 5-HETE could induce the expressions of MMPs such as MMP-2 and MMP-23, and thus contribute to the degradation of matrix components and exacerbate VR in AMI. On the other hand, increased expression of ALOX5AP accelerates the accumulation of LTA4 and thus enhances atherosclerosis. Evidently, QSYQ treatment can effectively suppress this LOX pathway by significant down-regulation of a number of key genes such as ALOX15, ALOX5AP, GPXs, MMP-2 and MMP-23.

COX (is also called prostaglandin synthase) is a kind of key enzyme in prostaglandin synthesis during AA metabolism. COX activation produces prostaglandin H2, which is subsequently converted to PGE2, PGF2α and PGI2 by the catalysis of PTGES2, FAM213B and PTGIS enzymes, respectively. It is note that these enzyme genes revealed significantly differential expression in our analyses. The expression of PTGIS in AMI model was increased compared with that in Sham-operated group. PGI2 has a cardioprotective effect by inhibiting platelet aggregation . Therefore, up-regulation of PTGIS expression in AMI progression may be indicative of a defend mechanism acting to prevent damage from myocardial infarct in AMI patients. Under the treatment of QSYQ, PTGES2 expression of QSYQ-treated was significantly up-regulated compared with AMI model group. While, PGE2 and its receptors are considered better targets with protective effects in curing heart failure in AMI . Especially the PGE2/EP4 (E-series prostanoid receptor 4) signaling is thought to be the potential targets to heart failure and myocardial ischemia in AMI . Moreover, it has been reported that PGE2 has actions that are potently anti-inflammatory . In studies with gastric epithelial cells, PGE2 inhibited ERK (MAPK) activation . Interestingly, several MAPKs, even their up-stream kinase genes were significantly down-regulated induced by QSYQ treatment, including MAPK3, MAPK14, MAP2K3, MAP3K4, MAP4K2 and MAP4K4. Hence, we could postulated that under the treatment of QSYQ on AMI, the significantly enhanced expression of PTGES2 results in the increment of PGE2. PGE2 exerts its anti-inflammatory action via inhibiting the activation of MAPKs and their up-stream kinases. Besides PTGES2, the expression of FAM213B was slightly up-regulated with 1.96 fold change and 0.04 P value but 0.12 FDR, in QSYQ-treated compared with AMI model group. Moreover, the catalysis product PGF2α also has important anti-inflammatory effects especially in the late phase of inflammation resolution .

As a kind of cytochrome, CYP2J10 was found to be markedly up-regulated after QSYQ treatment. CYP2J10 catalyzes the conversions of AA to 19(S)-HETE and some epoxyeicosatrienoic acids (EETs), the conversion of 15(S)-HPETE to some hydroxyl epoxyeicosatrienoic acid (EETAs) and trihydroxyicosatrienoic acid (THETAs). As one of metabolites of AA, 14, 15-EETs have beneficial effects in certain cardiovascular diseases. The increased 14, 15-EET levels protect the cardiovascular system, including vasodilation, angiogenesis, decreasing platelet aggregation, and generally acting to maintain vascular homeostasis . More importantly, EETs have anti-inflammatory effects that play important roles in the prevention of AMI . Interestingly, EETs also inhibit the expression of vascular cell adhesion molecule-1 in human endothelial cells , which supports our results. Our results also highlighted the suppressed expression of vascular cell adhesion molecule 1 (VCAM1) after QSYQ treatment, which may suggested the increment of EETs induced by CYP2J10 inhibited the expression of VCAM1 and thus attenuated inflammation. Moreover, it has been reported that EETs exert many other cardioprotective effects, including the reduction of myocardial stunning, myocardial infarct size, and inflammatory response; prevention of the onset of left ventricular hypertrophy and subsequent remodeling, which leads to heart failure; and reduction of the incidence of cardiac arrhythmias associated with heart failure in AMI .

**Figure S7. Diagram of significant expression changes of enzyme genes during overall metabolism of fatty acid in AMI progression and under the treatment of QSYQ.**


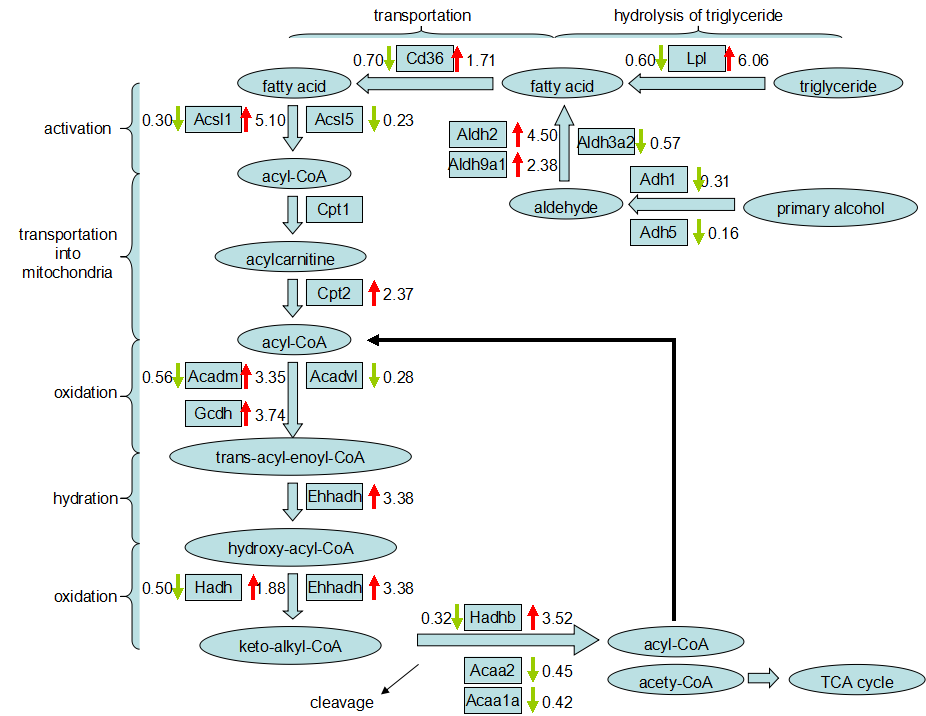


Figure S7. Diagram of significant expression changes of enzyme genes during overall metabolism of fatty acid in AMI progression and under the treatment of QSYQ. For one gene, left arrow and number indicate fold change for comparison of AMI model with Sham-operated group; right arrow and number represent fold change for comparison of QSYQ-treated with AMI model group.

The initial step of fatty acid metabolism is hydrolysis of triglyceride (TG). As shown in Figure S7, our results revealed that Lipoprotein lipase (LPL), which encodes lipoprotein lipase and is expressed in heart, muscle, and adipose tissue, had significantly lower expression in AMI progression and inversely enhanced after QSYQ treatment. LPL plays an important role in hydrolysis of core TGs of chylomicrons and very low-density lipoprotein (VLDL) and enhances the high density lipoprotein cholesterol (HDL-C) level by processing HDL to its mature form . It is note that the increasing concentrations of HDL-C are strongly associated with decreasing accumulation of atherosclerosis within the walls of arteries over weeks, years, decades. While LDL particles are often called bad cholesterol or unhealthy cholesterol, because they deliver fat molecules to macrophages in the wall of arteries. Thus, the significantly enhanced expression of LPL under the treatment of QSYQ may contribute to higher concentration of HDL-C in AMI progression.

After hydrolysis of TG into long chain fatty acids, the next step is the transportation of fatty acids to cytoplasm from extracelluar environment. CD36 as a fatty acid translocase, binds and transports long chain fatty acids. CD36 was decreased in AMI progression and to be inversely increased after QSYQ treatment. Previous study has suggested possible protective effects of higher soluble CD36 in CAD patients. Meanwhile higher soluble CD36 concentration is also associated with lower risk of left ventricular hypertrophy. Thus, our results indicated that overexpression of CD36 induced by QSYQ may be associated with lower risk of left ventricular hypertrophy.

Once transported into cytoplasm, long chain fatty acids participate in several steps of degradation. Fatty acid degradation is the process in which fatty acids are broken down into their metabolites, and generates acetyl-CoA. Acetyl-CoA is the entry molecule for the citric acid cycle and the main energy supply of animals. It includes 3 major steps: activation, transport into mitochondria and β-oxidation. Once fatty acid is inside the mitochondria, β-oxidation of fatty acid occurs via 5 recurring steps: activation by ATP, oxidation by FAD, hydration, oxidation by NAD+, thiolysis and the final product is acetyl-CoA.

Firstly, at the step of activation of fatty acids, 2 long-chain acyl-CoA synthetases (ACSL1 and ACSL5) which catalyze long-chain fatty acids conversion to acyl-CoAs, was demonstrated markedly gene expression changes in our study. ACSL1 was significantly reduced in AMI progression and reversely enhanced after QSYQ treatment. While ACSL5 was down-regulated after QSYQ treatment. ACSL1 is a membrane-associated enzyme presenting on the mitochondria and endoplasmic reticulum, which catalyzes the activation of long-chain fatty acids to form acyl-CoAs . ACSL1 is highly expressed in oxidative tissues like brown adipose tissue and heart, and depleting cardiac ACSL1 expression results in a 90% decrease in the mitochondrial oxidation of long-chain acyl-CoAs . Activated fatty acids normally provide 60% to 90% of the substrate used by the heart for energy, but when ACSL1 is absent, fatty acids oxidation decreases more than 90% . Endogenous ACSL1 in heart directs its acyl-CoA product towards β-oxidation rather than towards the synthesis of complex lipids . More interestingly, several gene expression changes are reported to be related with ACSL1. AMPK activity is observed to be down-regulated in ACSL1-deficient hearts (ACSL1 H^−/−^ hearts). AMP-activated protein kinase β 1 non-catalytic subunit (PRKAB1), as one of AMPKs was significantly down-regulated in AMI progression and reversed to be enhanced expressed after QSYQ treatment, which share the same gene expression change with ACSL1. Moreover, the expression of glutathione S-transferase alpha 1 (GSTA1) is reported to be 15-fold higher in ACSL1 H^−/−^ hearts than controls . Of note, our results showed that GSTA1 was overexpressed in AMI progression and inhibited after QSYQ treatment, which was in agreement with previous study. Hence, we postulate that the deficient gene expression of ACSL1 may contribute to the down-regulation of PRKAB1 and overexpression of GSTA1 in hearts in AMI progression. Besides ACSL1, another long-chain acyl-CoA synthetase isoform ACSL5, was repressed after QSYQ treatment. However, ACSL5 is highly expressed in uterus and spleen, and in trace amounts in normal brain, but has markedly increased levels in malignant gliomas. And expression of ACSL5 was evidently lower than ACSL1 in hearts as observation in our results and the significant up-regulation of ACSL1 was seem to be predominant in treatment of QSYQ.

At the second step of fatty acid degradation, carnitine palmitoyltransferase 2 (CPT2) together with carnitine palmitoyltransferase 1 (CPT1), controls the rate of long-chain acyl-CoA transport into the mitochondria from cytoplasm. CPT2 was overexpressed after QSYQ treatment, which promotes fatty acid β-oxiadation in mitochondria.

Once activated and transported into mitochondrial matrix, fatty acids participate in β-oxidation in the mitochondrial matrix space. Acyl-CoA dehydrogenases such as ACADM and ACADVL, catalyze the initial step of the mitochondrial fatty acid β-oxidation pathway — the conversion of acyl-CoA into trans-2, 3-dehydroacyl-CoA. The ACADM (acyl-CoA dehydrogenase, C-4 to C-12 straight chain) gene encodes the medium-chain specific (C4 to C12 straight chain) acyl-coenzyme A dehydrogenase. Our results indicated that QSYQ up-regulated expression of ACADM and reverse the abnormal changes in the expression in AMI progression, which is lent supported by previous studies. Van *et al*. have shown that at the level of the skeletal muscle, chronic heart failure patients are characterized by decreased expression of metabolic genes including ACADM , which is in keeping with our results. On the other hand, ACADVL was down-regulated after QSYQ treatment. But, the ACADVL is specific to long-chain and very-long-chain fatty acids such as palmitoyl-CoA, mysritoyl-CoA and stearoyl-CoA. It is noteworthy that ACADVL can accommodate substrate acyl chain whose length is as long as 24 carbons, but shows little activity for substrates of less than 12 carbons. Thus, the significant up-regulation of ACADM seemed to not only compensate the decreased expression of ACADVL, but also effectively promote the initial step of the mitochondrial fatty acid β-oxidation pathway.

After dehydrogenation of acyl-CoA by acyl-CoA dehydrogenases, the next step is the hydration of the bond between C-2 and C-3 and to produce L-β-hydroxyacyl CoA. As our results shown, EHHADH which encodes a bifunctional enzyme involved in hydration of β-oxidation, was overexpressed after QSYQ treatment. The N- terminal region of EHHADH contains enoyl-CoA hydratase activity while the C-terminal region contains 3-hydroxyacyl-CoA dehydrogenase activity.

While at the step of the oxidation of L-β-hydroxyacyl CoA by NAD^+^, hydroxyl group of fatty acid is converted into a keto group. Two hydroxyacyl-CoA dehydrogenase genes including HADH and EHHADH, were enhanced after QSYQ treatment. In the meantime, HADH was down-regulated in AMI progression. HADH is a member of the 3-hydroxyacyl-CoA dehydrogenase gene family, which functions in the mitochondrial matrix to catalyze the oxidation of straight-chain 3-hydroxyacyl-CoAs in β-oxidation pathway. Its enzymatic activity is highest with medium-chain-length fatty acids and is an allosteric enzyme of the fatty acid β-oxidation pathway .

The final step is the cleavage of β-ketoacyl CoA by the thiol group of another molecule of coenzyme A. Three 3-ketoacyl-CoA thiolase genes including HADHB, ACAA2 and ACAA1A had significantly differential expression changes after QSYQ treatment. Among them, HADHB was down-regulated in AMI progression and reserved to be enhanced after QSYQ treatment. The HADHB (hydroxyacyl-CoA dehydrogenase/3-ketoacyl-CoA thiolase/enoyl-CoA hydratase (trifunctional protein), β subunit) gene encodes the β subunit of the mitochondrial trifunctional protein (MTP), which catalyzes the last three steps of mitochondrial β-oxidation of long chain fatty acids.

In addition to fatty acid oxidation enzyme genes, 2 alcohol dehydrogenases (ADH1 and ADH5) were suppressed while expressions of 2 aldehyde dehydrogenases including ALDH2 and ALDH9A1 were enhanced after QSYQ treatment. Such gene expression changes result in the decrease in conversion of alcohol into aldehyde and inverse increase in conversion of aldehyde into fatty acid, which may diminish the cardiotoxicity of aldehyde in AMI progression.

**Figure S8. Diagram of gene expression changes of ECM components and integrins.**


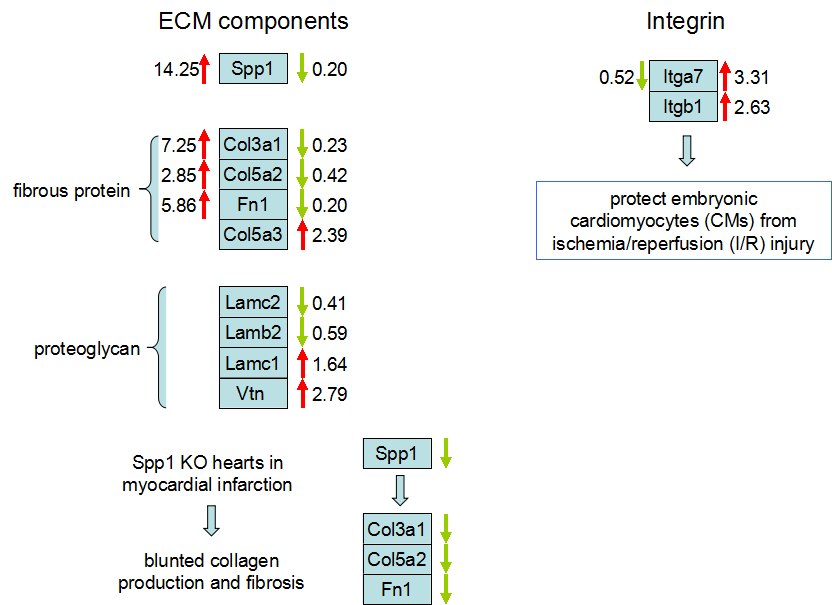


Figure S8. Diagram of gene expression changes of ECM components and integrins. For one gene, left arrow and number indicate fold change for comparison of AMI model with Sham-operated group; right arrow and number represent fold change for comparison of QSYQ-treated with AMI model group.

Extracellular matrix (ECM) in the heart and vascular wall consists of fibrous proteins and proteoglycans . These ECM components are important for maintenance of both the structure and function of the heart and vascular tissues . In our study, several ECMs were elevated in AMI progression and revealed reverse down-regulated expression changes after QSYQ treatment, which were supported by previous studies. These ECMs included secreted phosphoprotein 1 (SPP1), secreted phosphoprotein 1 (FN1), collagen type III α 1 (COL3A1), collagen type III α1 (COL5A2), laminin gamma 2 (LAMC2), laminin β2 (LAMB2) and reelin (RELN). Besides these ECMs, collagen type V α 3 (COL5A3), laminin gamma 1 (LAMC1) and vitronectin (VTN) had higher expressions after QSYQ treatment. It has been reported that in atherosclerotic lesions, the levels of ECM components, particularly fibrillar collagen such as COL3A1 and COL5A2, are elevated . Besides fibrillar collagen, SPP1 is also reported to be markedly up-regulated in the left anterior descending coronary artery (LAD) of obese pigs are implicated in atherosclerosis . In addition, SPP1 is reported to be involved in hypertrophic and fibrotic responses of the heart . Of note, the increased expression of ECM including collagen and FN1 are hallmarks of cardiac fibrosis . Additionally, it has been reported that FN is a target of TGFβ signaling and the expression of FN1 is known to be up-regulated by TGFβ in human fibroblast culture . Interestingly, it has also been reported that the down-regulation of TGFβ2 in the avian heart forming region embryo causes a decrease in FN expression. Given that TGFβ3 as one of TGFβs, was significantly up-regulated in AMI progression and inhibited after QSYQ treatment, we could postulate that the enhanced expression of TGFβ3 induce increased expression of FN1 in AMI progression, and after treatment of QSYQ, the expression of TGFβ3 was down-regulated and hence caused inhibition of FN1. More importantly, as shown in study from Singh’s group, SPP1 KO hearts had a reduced hypertrophic response following aortic constriction, and also showed blunted collagen production and fibrosis following myocardial infarction , which lends supports to our results of concordant down-regulated expressions of COL3A1, COL5A2, FN1 and SPP1. In summary, our results indicated that QSYQ could effectively reverse the abnormal changes in the activities of ECM components in rats after myocardial infarction (MI).

Integrins are a family of transmembrane heterodimeric receptors composed of α and β subunits that serve as the primary link between ECM ligands and cytoskeletal structures . Integrin β1 is recognized to have 4 splice variants. The β1D variant is expressed dominantly in striated muscles, including the heart. Interestingly, work has shown that the myocyte β1D integrin form is down-regulated in the myocardium after myocardial infarction (MI). This process has been suggested to play an important role in the decreased function of the heart after MI by reducing the ability of the embryonic cardiomyocytes (CMs) to interact appropriately with the ECM . In addition, Krishnamurthy *et al.* have reported that mice globally deficient in β1 integrin (heterozygous integrin β1 knockout mice) had worsened myocardial function after MI compared with that in control animals, with increased apoptosis . ITGB1 (integrin, β1) was up-regulated in rats after QSYQ-treated group, which suggested that QSYQ could improve myocardial function after MI by enhancing ITGB1 expression. ITGB1 partners with several α integrin subunits in the myocyte, including α7. Integrin α7 (ITGA7) partners with β1 to form a major laminin-binding (LN-binding) receptor that is highly expressed in the adult myocyte. The gene expression of ITGA7 was decreased in AMI progression and reversely overexpressed after treatment of QSYQ. More importantly, Hideshi *et al.* have found that α7β1D integrin can protect embryonic cardiomyocytes (CMs) from ischemia/reperfusion (I/R) injury . More specifically, Hideshi *et al.* have found that α7β1D preserved mitochondrial membrane potential during hypoxia/reoxygenation (H/R) injury via inhibition of mitochondrial Ca^2+^ overload but did not alter H/R effects on oxidative stress. And they have suggested that α7β1D integrin modifies Ca^2+^ regulatory pathways and offers a means to protect the myocardium from ischemic injury. Thus, the coincident increased expressions of ITGA7 and ITGB1 as shown in our data demonstrated that QSYQ protects the myocardium from ischemic injury by α7β1 integrin.

**Figure S9. Diagram of significant expression changes of abundant enzyme genes involved in branched-chain amino acids (BCAAs) degradation.**


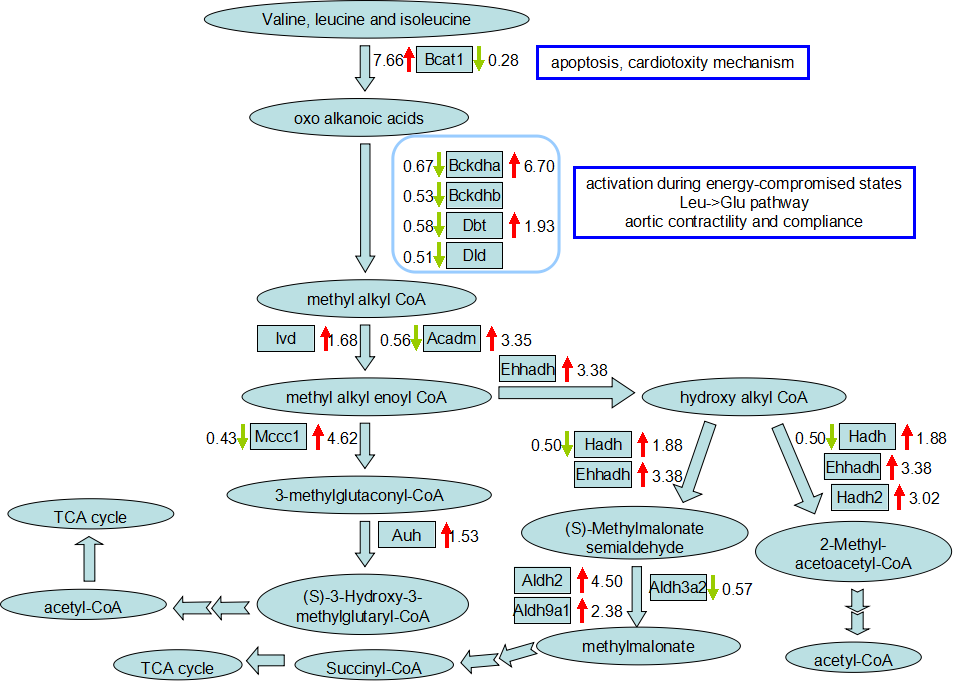


Figure S9. Diagram of significant expression changes of abundant enzyme genes involved in branched-chain amino acids (BCAAs) degradation. For one gene, left arrow and number indicate fold change for comparison of AMI model with Sham-operated group; right arrow and number represent fold change for comparison of QSYQ-treated with AMI model group.

Valine, leucine and isoleucine are the only branched-chain amino acids (BCAAs) among proteinogenic amino acids and they appear to have anabolic effects on protein metabolism in muscle tissue. BCAAs are oxidative energy substrates for the heart and exert anabolic effects on myocardial protein . It has been reported that BCAAs are elevated in heart failure . BCAA homeostasis is controlled by mitochondrial branched-chain alpha-keto acid dehydrogenase complex (BCKDC), which participates in degradation of BCAAs. In humans, BCAAs are found at high levels in skeletal muscle, where they contribute to energy production by catabolism after the activation of BCKDC during energy-compromised states such as, exercise or fever . Four genes encode the catalytic subunits of BCKDC: E1α (BCKDHA), E1β (BCKDHB), E2 (DBT), and E3 (DLD) . Interestingly, all of these 4 genes were detected to be significantly deferentially down-regulated in AMI model rats and after treatment of QSYQ, among them, 2 genes including BCKDHA (branched chain keto acid dehydrogenase E1, alpha polypeptide) and DBT (dihydrolipoamide branched chain transacylase E2), which encodes the α subunit of the decarboxylase (E1) component and transacylase (E2) subunit respectively, were revealed pronounced overexpression. A deficiency in any of the enzymes of this complex as well as an inhibition of the complex as a whole leads to a buildup of branched-chain amino acids and their harmful derivatives in the body . More importantly, it has been reported that the biochemical route of the leucine-to-glumate (Leu->Glu) pathway is metabolism of leucine to acetyl CoA. And the controlling reaction of the pathway is mediated by the BCKDC, and that glutamate formation via the Leu->Glu pathway is a major source of aortic segment free glutamate in vitro . In addition, the results of Schachter et al. support the hypothesis that the Leu->Glu pathway functions in the regulation of aortic contractility and compliance. Thus, we could make the hypothesis that the deficiency of the BCKDC not only decrease the degradation of BCAAs and then contribute to the compromise of energy in AMI progression, but also influence cardiovascular contractility and compliance. Enhancement of BCKDC expression after the treatment of QSYQ accelerates the metabolism of BCAAs and effectively promotes the transformation of leucine to glutamate, which can contribute to regulation of cardiovascular contractility and compliance.

BCAT1 encodes the cytosolic form of the enzyme branched-chain amino acid transaminase which catalyzes the transamination of branched-chain L-amino acids (BCAAs) to corresponding oxo-alkanoic acids including 4-methyl-2-oxopentanoate, 3-methyl-2-oxobutanoic acid and (S)-3-Methyl-2-oxopentanoic acid. BCAT1 was elevated in AMI progression and suppressed after treatment of QSYQ in our study. In addition, BCAT1 plays a key role in apoptosis, which is known to play a major role in the cardiotoxity mechanism . Thus, the attenuation of BCAT1 under the treatment of QSYQ drug may be involved in suppressing cardiotoxity in AMI progression.

In addition to the distinct reversed expression change of BCKDC, most enzyme genes including ACADM, MCCC1 and HADH involved in BCAAs degradation were suppressed in AMI model group and have significantly differential up-regulated expression in rats after QSYQ treatment. The ACADM (acyl-CoA dehydrogenase, C-4 to C-12 straight chain) gene encodes the medium-chain specific (C4 to C12 straight chain) acyl-Coenzyme A dehydrogenase. Our results indicated that QSYQ could reverse the abnormal changes in expression of ACADM, which is supported by previous studies. Van *et al*. have shown that at the level of the skeletal muscle, chronic heart failure patients are characterized by downstream metabolic genes including ACADM , which is in keeping with our results. The MCCC1 (methylcrotonoyl-CoA carboxylase 1) gene encodes the large subunit of 3-methylcrotonyl-CoA carboxylase, which functions as a heterodimer and catalyzes the carboxylation of 3-methylcrotonyl-CoA to form 3-methylglutaconyl-CoA in the leucine catabolic pathway. It is recommended that MCCC1 deficiency should be included in the differential diagnosis of dilative cardiomyopathy , which is indicated the association between deficiency of MCCC1 and pathogenesis of cardiomyopathy. The HADH (hydroxyacyl-CoA dehydrogenase) is a member of the 3-hydroxyacyl-CoA dehydrogenase gene family, which functions in the mitochondrial matrix to catalyze the oxidation of straight-chain 3-hydroxyacyl-CoAs as part of the β-oxidation pathway. Its enzymatic activity is highest with medium-chain-length fatty acids and is an allosteric enzyme of the fatty acid β-oxidation pathway . The HADHB (hydroxyacyl-CoA dehydrogenase/3-ketoacyl-CoA thiolase/enoyl-CoA hydratase (trifunctional protein), β subunit) encodes the β subunit of the mitochondrial trifunctional protein (MTP), which catalyzes the last three steps of mitochondrial β-oxidation of long chain fatty acids. MTP complex is composed of 4 hydroxyacyl-CoA dehydrogenase-alpha (HADHA) and 4 hydroxyacyl-CoA dehydrogenase-β (HADHB) subunits, which catalyze the last 3 steps in the fatty acid β-oxidation spiral of long-chain fatty acids. Besides these 3 important enzyme genes, several other enzyme genes including isovaleryl-CoA dehydrogenase (IVD), enoyl-CoA, hydratase/3-hydroxyacyl CoA dehydrogenase (EHHADH), AU RNA binding protein/enoyl-CoA hydratase (AUH), hydroxysteroid (17-β) dehydrogenase 10 (HSD17B10), 3-hydroxy-3-methylglutaryl-CoA synthase 1 (HMGCS1), aldehyde dehydrogenase 2 family (ALDH2) and aldehyde dehydrogenase 9 family, member A1 (ALDH9A1) were markedly up-regulated in rats after QSYQ treatment.

Abundant enzyme genes involved in BCAAs degradation were significantly differential up-regulated and thus accelerated metabolism of BCAAs. BCAAs are reported to be independently correlated with increased carotid intima-media thickness (cIMT), which is one of important risk factors of CAD . In addition, BCAA can be used as an oxidative fuel by heart which has high levels of BCAA transaminase and BCKDC . Thus, the acceleration of BCAA metabolism can contribute to regulation of cardiomyocyte contractility and compliance, alleviation of cardiotoxity in AMI progression and provide an oxidative fuel by heart.

**Table S1 Description of the top 20 keystone QSYQ-regulated genes.**

Table S1 Description of the top 20 keystone QSYQ-regulated genes.

| Gene symbol | PageRank | Function | Summary^#^ |
| --- | --- | --- | --- |
| RPL22 | 0.00334 | Encodes a cytoplasmic ribosomal protein that is a component of the 60S subunit | RNA binding |
| ALDOA | 0.00314 | A glycolytic enzyme that catalyzes the reversible conversion of fructose-1,6-bisphosphate to glyceraldehyde 3-phosphate and dihydroxyacetone phosphate | Glycolysis and gluconeogenesis |
| PCM1 | 0.00308 | A component of centriolar satellites, which are electron dense granules scattered around centrosomes | Cell cycle and mitotic |
| SCAMP2 | 0.00302 | As carriers to the cell surface in post-golgi recycling pathways | Post-Golgi recycling |
| DYSF | 0.00299 | Involved in muscle contraction and contains C2 domains that play a role in calcium-mediated membrane fusion events | Calcium-dependent phospholipid binding |
| PRSS23 | 0.00297 | Encodes a conserved member of the trypsin family of serine proteases | Serine-type endopeptidase activity and extracellular matrix |
| SGK1 | 0.00296 | Encodes a serine/threonine protein kinase that plays an important role in cellular stress response | Protein serine/threonine kinase activity and sodium channel regulator activity |
| SLC6A8 | 0.00295 | A plasma membrane protein whose function is to transport creatine into and out of cells | Neurotransmitter:sodium symporter activity and creatine transmembrane transporter activity |
| TBRG1 | 0.00294 | Acts as a growth inhibitor, activates p53/TP53, causes G1 arrest and collaborates with CDKN2A to restrict proliferation | NF-KappaB family pathway and extracellular matrix |
| GPX7 | 0.00292 | Protects esophageal epithelia from hydrogen peroxide-induced oxidative stress, suppresses acidic bile acid-induced reactive oxigen species (ROS) and protects against oxidative DNA damage and double-strand breaks. | Glutathione peroxidase activity |
| CYB561D2 | 0.00287 | Cytochrome B561 Family, Member D2 | Metal ion binding |
| CDK1 | 0.00287 | A catalytic subunit of the highly conserved protein kinase complex known as M-phase promoting factor (MPF), which is essential for G1/S and G2/M phase transitions of eukaryotic cell cycle | Protein kinase activity and RNA polymerase II carboxy-terminal domain kinase activity |
| TGFB1I1 | 0.00284 | Functions as a molecular adapter coordinating multiple protein-protein interactions at the focal adhesion complex and in the nucleus, links various intracellular signaling modules to plasma membrane receptors and regulates the Wnt and TGFB signaling pathways | Transcription coactivator activity and extracellular matrix |
| IGFBP7 | 0.00282 | Binds IGF-I and IGF-II with a relatively low affinity and stimulates prostacyclin I2 (PGI2) production, stimulates cell adhesion. | Insulin-like growth factor binding and extracellular matrix |
| ABHD17A | 0.00282 | Abhydrolase Domain Containing 17A | Hydrolase activity |
| LOXL3 | 0.00282 | Both isoforms function as amine oxidases toward elastin and different types of collagens, Isoform 1 shows the highest activity toward collagen type VIII, while Isoform 2 presents the highest activity toward collagen type IV | Degradation of the extracellular matrix and elastic fibre formation |
| LTBP4 | 0.00282 | Binds transforming growth factor beta (TGFB) as it is secreted and targeted to the extracellular matrix | Calcium ion binding and extracellular matrix |
| LITAF | 0.00282 | Encodes lipopolysaccharide-induced TNF-alpha factor, which is a DNA-binding protein and can mediate the TNF-alpha expression by direct binding to the promoter region of the TNF-alpha gene | Signal transducer activity and extracellular matrix |
| PDIA5 | 0.00281 | Catalyzes the rearrangement of -S-S- bonds in proteins | Electron carrier activity and protein disulfide oxidoreductase activity |
| CMTM7 | 0.00281 | One of several chemokine-like factor genes located in a cluster on chromosome 3 | Cytokine activity |

^#^The information about the genes was adopted from www.genecards.org. Genes were annotated according to their functions.

ALDOA and SLC6A8 are involved in energy metabolism. ALDOA encodes fructose bisphosphate aldolase A, which is involved in glycolysis. SLC6A8 is a creatine transporter (CrT) that accomplishes Na^+^ coupled cellular uptake of creatine in several tissues including kidney, intestine, heart, skeletal muscle and brain . It was found that QSYQ treatment significantly reversed the decreased expressions both of ALDOA and SLC6A8 in AMI. Of note, studies in mice with genetic overexpression of the CrT demonstrate proof-of-principle that elevated creatine protects the heart from ischaemia-reperfusion (IR) injury . This suggests the activation of SLC6A8 acts as a major pharmacological target of QSYQ treatment on IR injury of AMI. Given that the heart has a very high energy demand, with more than 95% of this energy demand being met by adenosine triphosphate (ATP) derived from mitochondrial oxidative phosphorylation and the rest from glycolysis . Thus, the increased glycolysis could make up the majority of the remaining contribution of cardiac energy supply.

**Table S2 Significance analysis for 15 functional groups.**

Table S2 Significance analysis for 15 functional groups.

| Number of group | Enrichment score (ES) | P value | Adjusted P value | Involved KEGG pathways |
| --- | --- | --- | --- | --- |
| **Group 1** | 4.162 | <0.0001 | <0.0001 | Glutathione metabolism, Arachidonic acid metabolism |
| **Group 2** | 3.528 | <0.0001 | <0.0001 | Pyruvate metabolism, Citrate cycle (TCA cycle) |
| **Group 3** | 3.093 | <0.0001 | <0.0001 | Valine, leucine and isoleucine degradation, Fatty acid degradation |
| **Group 4** | 2.886 | <0.0001 | <0.0001 | Focal adhesion, ECM-receptor interaction |
| **Group 5** | 2.397 | <0.0001 | <0.0001 | Pentose phosphate pathway, Glycolysis / Gluconeogenesis |
| **Group 6** | 1.601 | 0.0002 | 0.0020 | Arginine and proline metabolism, Alanine, aspartate and glutamate metabolism |
| Group 7 | 1.479 | 0.0072 | 0.0576 | Focal adhesion, Regulation of actin cytoskeleton |
| Group 8 | 1.299 | 0.0008 | 0.0072 | Phagosome, Gap junction |
| Group 9 | 1.131 | 0.0105 | 0.0636 | Adrenergic signaling in cardiomyocytes, Endocrine and other factor-regulated calcium reabsorption |
| Group 10 | 1.105 | 0.0106 | 0.0636 | Platelet activation, Chemokine signaling pathway |
| Group 11 | 0.551 | 0.0566 | 0.2830 | Insulin signaling pathway, Focal adhesion |
| Group 12 | 0.474 | 0.1431 | 0.4293 | Adrenergic signaling in cardiomyocytes, Cardiac muscle contraction |
| Group 13 | 0.368 | 0.0843 | 0.3372 | RNA degradation |
| Group 14 | -0.021 | 1.0000 | 1.0000 | Renin secretion |
| Group 15 | -0.021 | 1.0000 | 1.0000 | Vascular smooth muscle contraction |

The functional groups are prioritized with their enrichment scores. Only functional groups with adjusted significance P value less than 0.005 are identified as significant functional groups and highlighted with bold.

**Table S3 Key genes of significant functional group and top keystone QSYQ-regulated genes.**

Table S3 Key genes of significant functional group and top keystone QSYQ-regulated genes.

| Gene symbol  (Entrez ID) | FDR  (AMI model vs Sham-operated) | Fold Change# (AMI model vs Sham-operated) | FDR (QSYQ-treated vs AMI model) | Fold Change# (QSYQ-treated vs AMI model) | Significant functional group |
| --- | --- | --- | --- | --- | --- |
| ALOX15 (81639) | 4.00e-05 | 9.58 | 0.08 | 0.08 | Group 1 |
| ALOX5AP (29624) | 4.34e-05 | 6.73 | 4.31e-07 | 0.20 | Group 1 |
| MMP2  (81686) | 5.17e-08 | 3.57 | 5.88e-21 | 0.17 | Group 1 |
| MMP23 (94339) | 7.55e-04 | 8.03 | 4.04e-05 | 0.12 | Group 1 |
| NOS3 (24600) | 0.20 (P value)* | 0.29 | 0.03 | 14 | Group 6 |
| LPL (24539) | 2.58e-03 | 0.60 | 1.24e-21 | 6.06 | Group 3 |
| CD36 (29184) | 0.07 | 0.70 | 7.32e-03 | 1.71 | Group 3 |
| ACSL1 (25288) | 2.05e-20 | 0.30 | 3.36e-15 | 5.10 | Group 3 |
| CPT2 (25413) | 0.20 | 0.77 | 4.46e-03 | 2.37 | Group 3 |
| ACADM (24158) | 4.69e-04 | 0.56 | 4.16e-08 | 3.35 | Group 3 |
| GCDH (364975) | 0.38 | 0.79 | 4.10e-07 | 3.74 | Group 3 |
| EHHADH (171142) | 0.12 (P value)* | 0.52 | 0.05 | 3.38 | Group 3 |
| HADH (113965) | 1.80e-03 | 0.50 | 0.03 | 1.88 | Group 3 |
| HADHB (171155) | 2.01e-06 | 0.32 | 1.35e-06 | 3.52 | Group 3 |
| IGFBP7 (289560) | 4.57e-06 | 3.20 | 3.67e-25 | 0.15 | Top keystone QSYQ-regulated gene |
| LTBP4 (292734) | 0.07 | 1.87 | 2.62-e04 | 0.46 | Top keystone QSYQ-regulated gene |
| TGFB3 (25717) | 6.31e-04 | 6.05 | 0.01 | 0.35 | Top keystone QSYQ-regulated gene |
| TBRG1 (300521) | 0.05 | 2.15 | 2.08e-14 | 0.17 | Top keystone QSYQ-regulated gene |
| TGFB1I1 (84574) | 1.45e-06 | 4.83 | 8.52e-23 | 0.09 | Top keystone QSYQ-regulated gene |
| LITAF (65161) | 1.08e-03 | 3.11 | 6.43e-12 | 0.15 | Top keystone QSYQ-regulated gene |
| LOXL3 (312478) | 6.31e-03 | 3.20 | 1.60e-06 | 0.22 | Top keystone QSYQ-regulated gene |
| SPP1 (25353) | 1.30e-11 | 14.25 | 1.02e-04 | 0.20 | Group 4 |
| FN1 (25661) | 2.38e-06 | 5.86 | 5.37e-07 | 0.20 | Group 4 |
| COL3A1 (84032) | 7.74e-14 | 7.25 | 3.41e-10 | 0.23 | Group 4 |
| COL5A2 (85250) | 5.57e-03 | 2.85 | 1.19e-03 | 0.42 | Group 4 |
| BCKDHA (25244) | 0.07 | 0.67 | 2.55e-18 | 6.70 | Group 3 |
| BCKDHB (29711) | 9.06e-04 | 0.53 | 0.43 | 1.25 | Group 3 |
| DBT (29611) | 0.01 | 0.58 | 0.01 | 1.93 | Group 3 |
| DLD (298942) | 5.01e-03 | 0.51 | 0.31 | 1.47 | Group 3 |
| PRKAB1 (83803) | 0.06 | 0.53 | 1.01e-07 | 4.35 | Group 5 |
| ALDOA  (24189) | 1.43e-06 | 0.43 | 4.57e-10 | 3.10 | Group 5 |
| SUCLG1 (114597) | 0.06 | 0.41 | 1.69e-03 | 21.04 | Group 2 |
| SUCLA2 (361071) | 1.85e-04 | 0.42 | 1.53e-10 | 3.65 | Group 2 |
| CKM (24265) | 3.22e-04 | 0.52 | 1.03e-07 | 2.76 | Group 2 |
| CrT (50690) | 6.94e-03 | 0.57 | 4.60e-04 | 2.17 | Top keystone QSYQ-regulated gene |

*FDR calculated from DESeq2 is NA, due to slightly low expression of NOS3 in cardiac tissue. Thus, P value is provided instead of FDR here.

#Fold change is the ratio between raw expression means of two groups.

**Table S4 Summarization of therapeutic effects of QSYQ based on significant functional groups.**

Table S4 Summarization of therapeutic effects of QSYQ based on significant functional groups.

| Therapeutic effects of QSYQ | Involved groups | Involved biological processes | Validated key genes |
| --- | --- | --- | --- |
| QSYQ attenuates inflammation through suppressing arachidonic acid LOX pathway and elevating production of NO | Group 1, Group 6 | Arachidonic acid metabolism; Production of nitric oxide from arginine metabolism | ALOX15, MMP-2, NOS3 |
| QSYQ ameliorates dyslipidaemia through elevating CD36-CPT2-LPL fatty acid oxidation | Group 3 | Fatty acid degradation | CPT2, CD36, LPL |
| QSYQ alleviates VR progression | Group 4 | Alterations in ECMs and integrins that reflect the amelioration in ventricular remodeling | COL3A1, COL5A2 |

**References**

1. Amin P, Singh M, Singh K (2011) beta-Adrenergic Receptor-Stimulated Cardiac Myocyte Apoptosis: Role of beta1 Integrins. J Signal Transduct 2011:179057 doi:10.1155/2011/179057

2. Asbun J, Villarreal FJ (2006) The pathogenesis of myocardial fibrosis in the setting of diabetic cardiomyopathy. J Am Coll Cardiol 47:693-700 doi:10.1016/j.jacc.2005.09.050

3. Asztalos BF (2004) High-density lipoprotein metabolism and progression of atherosclerosis: new insights from the HDL Atherosclerosis Treatment Study. Curr Opin Cardiol 19:385-391 doi:10.1097/01.hco.0000126979.41946.7e

4. Conrad M, Sandin Å, Förster H, Seiler A, Frijhoff J, Dagnell M, Bornkamm GW, Rådmark O, van Huijsduijnen RH, Aspenström P (2010) 12/15-lipoxygenase–derived lipid peroxides control receptor tyrosine kinase signaling through oxidation of protein tyrosine phosphatases. Proceedings of the National Academy of Sciences 107:15774-15779 doi:10.1073/pnas.1007909107

5. Dixelius J, Jakobsson L, Genersch E, Bohman S, Ekblom P, Claesson-Welsh L (2004) Laminin-1 promotes angiogenesis in synergy with fibroblast growth factor by distinct regulation of the gene and protein expression profile in endothelial cells. J Biol Chem 279:23766-23772 doi:10.1074/jbc.M311675200

6. Drake KJ, Sidorov VY, McGuinness OP, Wasserman DH, Wikswo JP (2012) Amino acids as metabolic substrates during cardiac ischemia. Experimental Biology and Medicine 237:1369-1378 doi:10.1258/ebm.2012.012025

7. Ellis JM, Mentock SM, Depetrillo MA, Koves TR, Sen S, Watkins SM, Muoio DM, Cline GW, Taegtmeyer H, Shulman GI, Willis MS, Coleman RA (2011) Mouse cardiac acyl coenzyme a synthetase 1 deficiency impairs Fatty Acid oxidation and induces cardiac hypertrophy. Mol Cell Biol 31:1252-1262 doi:10.1128/MCB.01085-10

8. Feng B, Chakrabarti S (2012) miR-320 Regulates Glucose-Induced Gene Expression in Diabetes. ISRN Endocrinol 2012:549875 doi:10.5402/2012/549875

9. Fernández‐Guerra P, Birkler RI, Merinero B, Ugarte M, Gregersen N, Rodríguez‐Pombo P, Bross P, Palmfeldt J (2014) Selected reaction monitoring as an effective method for reliable quantification of disease-associated proteins in maple syrup urine disease. Molecular genetics & genomic medicine 2:383-392 doi:10.1002/mgg3.88

10. Fezai M, Elvira B, Borras J, Ben-Attia M, Hoseinzadeh Z, Lang F (2014) Negative regulation of the creatine transporter SLC6A8 by SPAK and OSR1. Kidney Blood Press Res 39:546-554 doi:10.1159/000368465

11. Funk CD (2001) Prostaglandins and leukotrienes: advances in eicosanoid biology. Science 294:1871-1875 doi:10.1126/science.294.5548.1871

12. Ghatpande SK, Zhou HR, Cakstina I, Carlson C, Rondini EA, Romeih M, Zile MH (2010) Transforming growth factor β2 is negatively regulated by endogenous retinoic acid during early heart morphogenesis. Development, growth & differentiation 52:433-455 doi:10.1111/j.1440-169X.2010.01183.x

13. Hedrick CC, Kim MD, Natarajan RD, Nadler JL (1999) 12-Lipoxygenase products increase monocyte:endothelial interactions. Adv Exp Med Biol 469:455-460 doi:10.1007/978-1-4615-4793-8_6

14. Hong D, Zeng X, Xu W, Ma J, Tong Y, Chen Y (2010) Altered profiles of gene expression in curcumin-treated rats with experimentally induced myocardial infarction. Pharmacol Res 61:142-148 doi:10.1016/j.phrs.2009.08.009

15. Honn KV, Timar J, Rozhin J, Bazaz R, Sameni M, Ziegler G, Sloane BF (1994) A lipoxygenase metabolite, 12-(S)-HETE, stimulates protein kinase C-mediated release of cathepsin B from malignant cells. Exp Cell Res 214:120-130 doi:10.1006/excr.1994.1240

16. Imig JD, Hammock BD (2009) Soluble epoxide hydrolase as a therapeutic target for cardiovascular diseases. Nature Reviews Drug Discovery 8:794-805 doi:10.1038/nrd2875

17. Ingwall JS, Weiss RG (2004) Is the failing heart energy starved? On using chemical energy to support cardiac function. Circulation research 95:135-145 doi:10.1161/01.RES.0000137170.41939.d9

18. Kelleher CM, McLean SE, Mecham RP (2004) Vascular extracellular matrix and aortic development. Curr Top Dev Biol 62:153-188 doi:10.1016/S0070-2153(04)62006-0

19. Krötz F, Riexinger T, Buerkle MA, Nithipatikom K, Gloe T, Sohn H-Y, Campbell WB, Pohl U (2004) Membrane potential-dependent inhibition of platelet adhesion to endothelial cells by epoxyeicosatrienoic acids. Arteriosclerosis, thrombosis, and vascular biology 24:595-600 doi:10.1161/01.ATV.0000116219.09040.8c

20. Krishnamurthy P, Subramanian V, Singh M, Singh K (2006) Deficiency of beta1 integrins results in increased myocardial dysfunction after myocardial infarction. Heart 92:1309-1315 doi:10.1136/hrt.2005.071001

21. Krzystolik A, Dziedziejko V, Safranow K, Kurzawski G, Rac M, Sagasz-Tysiewicz D, Poncyljusz W, Jakubowska K, Chlubek D, Rac ME (2015) Is plasma soluble CD36 associated with cardiovascular risk factors in early onset coronary artery disease patients? Scandinavian Journal of Clinical & Laboratory Investigation:1-9 doi:10.3109/00365513.2015.1031693

22. Magnusson LU, Lundqvist A, Asp J, Synnergren J, Johansson CT, Palmqvist L, Jeppsson A, Hulten LM (2012) High expression of arachidonate 15-lipoxygenase and proinflammatory markers in human ischemic heart tissue. Biochem Biophys Res Commun 424:327-330 doi:10.1016/j.bbrc.2012.06.117

23. Malan D, Reppel M, Dobrowolski R, Roell W, Smyth N, Hescheler J, Paulsson M, Bloch W, Fleischmann BK (2009) Lack of laminin gamma1 in embryonic stem cell-derived cardiomyocytes causes inhomogeneous electrical spreading despite intact differentiation and function. Stem Cells 27:88-99 doi:10.1634/stemcells.2008-0335

24. Manso AM, Kang SM, Ross RS (2009) Integrins, focal adhesions, and cardiac fibroblasts. J Investig Med 57:856-860 doi:10.231/JIM.0b013e3181c5e61f

25. McNulty PH, Jacob R, Deckelbaum LI, Young LH (2000) Effect of hyperinsulinemia on myocardial amino acid uptake in patients with coronary artery disease. Metabolism 49:1365-1369 doi:10.1053/meta.2000.9510

26. Merhi M, Demirdjian S, Hariri E, Sabbah N, Youhanna S, Ghassibe-Sabbagh M, Naoum J, Haber M, Othman R, Kibbani S, Chammas E, Kanbar R, Bayeh HE, Chami Y, Abchee A, Platt DE, Zalloua P, Khazen G (2015) Impact of inflammation, gene variants, and cigarette smoking on coronary artery disease risk. Inflamm Res 64:415-422 doi:10.1007/s00011-015-0821-1

27. Michaelis UR, Fisslthaler B, Medhora M, Harder D, Fleming I, Busse R (2003) Cytochrome P450 2C9-derived epoxyeicosatrienoic acids induce angiogenesis via cross-talk with the epidermal growth factor receptor (EGFR). FASEB J 17:770-772 doi:10.1096/fj.02-0640fje

28. Mitch WE (1980) Metabolism and metabolic effects of ketoacids. Am J Clin Nutr 33:1642-1648 doi:10.1002/dmr.5610050106

29. Moreno JJ (2009) New aspects of the role of hydroxyeicosatetraenoic acids in cell growth and cancer development. Biochem Pharmacol 77:1-10 doi:10.1016/j.bcp.2008.07.033

30. Node K, Huo Y, Ruan X, Yang B, Spiecker M, Ley K, Zeldin DC, Liao JK (1999) Anti-inflammatory properties of cytochrome P450 epoxygenase-derived eicosanoids. Science 285:1276-1279 doi:10.1126/science.285.5431.1276

31. Okada H, Lai NC, Kawaraguchi Y, Liao P, Copps J, Sugano Y, Okada-Maeda S, Banerjee I, Schilling JM, Gingras AR, Asfaw EK, Suarez J, Kang SM, Perkins GA, Au CG, Israeli-Rosenberg S, Manso AM, Liu Z, Milner DJ, Kaufman SJ, Patel HH, Roth DM, Hammond HK, Taylor SS, Dillmann WH, Goldhaber JI, Ross RS (2013) Integrins protect cardiomyocytes from ischemia/reperfusion injury. J Clin Invest 123:4294-4308 doi:10.1172/JCI64216

32. Padilla J, Jenkins NT, Lee S, Zhang H, Cui J, Zuidema MY, Zhang C, Hill MA, Perfield JW, 2nd, Ibdah JA, Booth FW, Davis JW, Laughlin MH, Rector RS (2013) Vascular transcriptional alterations produced by juvenile obesity in Ossabaw swine. Physiol Genomics 45:434-446 doi:10.1152/physiolgenomics.00038.2013

33. Pillinger MH, Marjanovic N, Kim S-Y, Scher JU, Izmirly P, Tolani S, Dinsell V, Lee Y-C, Blaser MJ, Abramson SB (2005) Matrix metalloproteinase secretion by gastric epithelial cells is regulated by E prostaglandins and MAPKs. Journal of Biological Chemistry 280:9973-9979 doi:10.1074/jbc.M413522200

34. Podebrad F, Heil M, Reichert S, Mosandl A, Sewell A, Böhles H (1999) 4, 5-dimethyl-3-hydroxy-2 [5h]-furanone (sotolone)—The odour of maple syrup urine disease. Journal of inherited metabolic disease 22:107-114 doi:10.1023/A:1005433516026

35. Ross RS (2004) Molecular and mechanical synergy: cross-talk between integrins and growth factor receptors. Cardiovascular research 63:381-390 doi:10.1016/j.cardiores.2004.04.027

36. Rowell LB, Shepherd JT (1996) Handbook of physiology: Section 12: Exercise: Regulation and integration of multiple systems. An American Physiological Society Book doi:10.5962/bhl.title.6400

37. Sankaralingam S, Lopaschuk GD (2015) Cardiac energy metabolic alterations in pressure overload–induced left and right heart failure (2013 Grover Conference Series). Pulmonary circulation 5:15 doi:10.1086/679608

38. Schachter D, Sang JC (2002) Aortic leucine-to-glutamate pathway: metabolic route and regulation of contractile responses. Am J Physiol Heart Circ Physiol 282:H1135-1148 doi:10.1152/ajpheart.00457.2001

39. Scher JU, Pillinger MH (2009) The anti-inflammatory effects of prostaglandins. J Investig Med 57:703-708 doi:10.231/JIM.0b013e31819aaa76

40. Schisler JC, Grevengoed TJ, Pascual F, Cooper DE, Ellis JM, Paul DS, Willis MS, Patterson C, Jia W, Coleman RA (2015) Cardiac energy dependence on glucose increases metabolites related to glutathione and activates metabolic genes controlled by mechanistic target of rapamycin. J Am Heart Assoc 4 doi:10.1161/JAHA.114.001136

41. Spiteller G (2006) Peroxyl radicals: inductors of neurodegenerative and other inflammatory diseases. Their origin and how they transform cholesterol, phospholipids, plasmalogens, polyunsaturated fatty acids, sugars, and proteins into deleterious products. Free Radical Biology and Medicine 41:362-387 doi:10.1016/j.freeradbiomed

42. Sprecher DL, Harris BV, Stein EA, Bellet PS, Keilson LM, Simbartl LA (1996) Higher triglycerides, lower high-density lipoprotein cholesterol, and higher systolic blood pressure in lipoprotein lipase-deficient heterozygotes. A preliminary report. Circulation 94:3239-3245 doi:10.1161/01.CIR.94.12.3239

43. Stanley WC, Recchia FA, Lopaschuk GD (2005) Myocardial substrate metabolism in the normal and failing heart. Physiol Rev 85:1093-1129 doi:10.1152/physrev.00006.2004

44. Stuewe SR, Gwirtz PA, Agarwal N, Mallet RT (2000) Exercise training enhances glycolytic and oxidative enzymes in canine ventricular myocardium. J Mol Cell Cardiol 32:903-913 doi:10.1006/jmcc.2000.1131

45. Stupack DG, Cheresh DA (2002) Get a ligand, get a life: integrins, signaling and cell survival. J Cell Sci 115:3729-3738 doi:10.1242/jcs.00071

46. Sun M, Opavsky MA, Stewart DJ, Rabinovitch M, Dawood F, Wen WH, Liu PP (2003) Temporal response and localization of integrins beta1 and beta3 in the heart after myocardial infarction: regulation by cytokines. Circulation 107:1046-1052 doi:10.1161/01.CIR.0000051363.86009.3C

47. Trueblood NA, Xie Z, Communal C, Sam F, Ngoy S, Liaw L, Jenkins AW, Wang J, Sawyer DB, Bing OH, Apstein CS, Colucci WS, Singh K (2001) Exaggerated left ventricular dilation and reduced collagen deposition after myocardial infarction in mice lacking osteopontin. Circ Res 88:1080-1087 doi:10.1161/hh1001.090842

48. Tso SC, Qi X, Gui WJ, Chuang JL, Morlock LK, Wallace AL, Ahmed K, Laxman S, Campeau PM, Lee BH, Hutson SM, Tu BP, Williams NS, Tambar UK, Wynn RM, Chuang DT (2013) Structure-based design and mechanisms of allosteric inhibitors for mitochondrial branched-chain alpha-ketoacid dehydrogenase kinase. Proc Natl Acad Sci U S A 110:9728-9733 doi:10.1073/pnas.1303220110

49. Tyagi SC (1999) Homocyst(e)ine and heart disease: pathophysiology of extracellular matrix. Clin Exp Hypertens 21:181-198 doi:10.3109/10641969909068660

50. Van Berendoncks AM, Garnier A, Beckers P, Hoymans VY, Possemiers N, Fortin D, Van Hoof V, Dewilde S, Vrints CJ, Ventura-Clapier R, Conraads VM (2011) Exercise training reverses adiponectin resistance in skeletal muscle of patients with chronic heart failure. Heart 97:1403-1409 doi:10.1136/hrt.2011.226373

51. Verrecchia F, Chu M-L, Mauviel A (2001) Identification of novel TGF-β/Smad gene targets in dermal fibroblasts using a combined cDNA microarray/promoter transactivation approach. Journal of Biological Chemistry 276:17058-17062 doi:10.1074/jbc.M100754200

52. Visser G, Suormala T, Smit GPA, Reijngoud D-J, Bink-Boelkens MTE, Niezen-Koning KE, Baumgartner ER (2000) 3-methylcrotonyl-CoA carboxylase deficiency in an infant with cardiomyopathy, in her brother with developmental delay and in their asymptomatic father. European journal of pediatrics 159:901-904 doi:10.1007/PL00008366

53. Wang Y, Li C, Liu ZY, Shi TJ, Wang QY, Li D, Wu Y, Han J, Guo SZ, Tang BH, Wang W (2014) DanQi Pill protects against heart failure through the arachidonic acid metabolism pathway by attenuating different cyclooxygenases and leukotrienes B4. Bmc Complem Altern M 14 doi:10.1186/1472-6882-14-67

54. Wang Y, Li C, Ouyang Y, Yu J, Guo S, Liu Z, Li D, Han J, Wang W (2012) Cardioprotective effects of Qishenyiqi mediated by angiotensin II type 1 receptor blockade and enhancing angiotensin-converting enzyme 2. Evidence-Based Complementary and Alternative Medicine 2012 doi:10.1155/2012/978127

55. Wang Y, Xu J, Chen J, Fan X, Zhang Y, Yu W, Liu J, Hui R (2013) Promoter variants of VTN are associated with vascular disease. Int J Cardiol 168:163-168 doi:10.1016/j.ijcard.2012.09.100

56. Xie Z, Singh M, Singh K (2004) Osteopontin modulates myocardial hypertrophy in response to chronic pressure overload in mice. Hypertension 44:826-831 doi:10.1161/01.HYP.0000148458.03202.48

57. Yang R, Dong J, Zhao H, Li H, Guo H, Wang S, Zhang C, Wang M, Yu S, Chen W (2014) Association of branched-chain amino acids with carotid intima-media thickness and coronary artery disease risk factors. PLoS One 9:e99598 doi:10.1371/journal.pone.0099598

58. Yang T, Peng R, Guo Y, Shen L, Zhao S, Xu D (2013) The role of 14,15-dihydroxyeicosatrienoic acid levels in inflammation and its relationship to lipoproteins. Lipids Health Dis 12:151 doi:10.1186/1476-511X-12-151

59. Ye Y-N, Liu ES-L, Shin VY, Wu WK-K, Cho C-H (2004) Contributory role of 5-lipoxygenase and its association with angiogenesis in the promotion of inflammation-associated colonic tumorigenesis by cigarette smoking. Toxicology 203:179-188

60. Zervou S, Whittington HJ, Russell AJ, Lygate CA (2015) Augmentation of creatine in the heart. Mini Rev Med Chem doi:10.2174/1389557515666150722102151
